# A Multicopper Oxidase-Related Protein Is Essential for Insect Viability, Longevity and Ovary Development

**DOI:** 10.1371/journal.pone.0111344

**Published:** 2014-10-20

**Authors:** Zeyu Peng, Peter G. Green, Yasuyuki Arakane, Michael R. Kanost, Maureen J. Gorman

**Affiliations:** 1 Department of Biochemistry and Molecular Biophysics, Kansas State University, Manhattan, Kansas, United States of America; 2 Department of Civil and Environmental Engineering, University of California Davis, Davis, California, United States of America; University of Cincinnati, United States of America

## Abstract

Typical multicopper oxidases (MCOs) have ten conserved histidines and one conserved cysteine that coordinate four copper atoms. These copper ions are required for oxidase activity. During our studies of insect MCOs, we discovered a gene that we named multicopper oxidase-related protein (MCORP). MCORPs share sequence similarity with MCOs, but lack many of the copper-coordinating residues. We identified MCORP orthologs in many insect species, but not in other invertebrates or vertebrates. We predicted that MCORPs would lack oxidase activity due to the absence of copper-coordinating residues. To test this prediction, we purified recombinant *Tribolium castaneum* (red flour beetle) MCORP and analyzed its enzymatic activity using a variety of substrates. As expected, no oxidase activity was detected. To study MCORP function *in vivo*, we analyzed expression profiles of TcMCORP and *Anopheles gambiae* (African malaria mosquito) MCORP, and assessed RNAi-mediated knockdown phenotypes. We found that both MCORPs are constitutively expressed at a low level in all of the tissues we analyzed. Injection of TcMCORP dsRNA into larvae resulted in 100% mortality prior to adult eclosion, with death occurring mainly during the pharate pupal stage or late pharate adult stage. Injection of TcMCORP dsRNA into pharate pupae resulted in the death of approximately 20% of the treated insects during the pupal to adult transition and a greatly shortened life span for the remaining insects. In addition, knockdown of TcMCORP in females prevented oocyte maturation and, thus, greatly decreased the number of eggs laid. These results indicate that TcMCORP is an essential gene and that its function is required for reproduction. An understanding of the role MCORP plays in insect physiology may help to develop new strategies for controlling insect pests.

## Introduction

The multicopper oxidase (MCO) family of enzymes is a large group of oxidases that share a similar structure but differ in substrate specificity [1]. Laccases, the largest subgroup of MCOs, can oxidize a broad range of aromatic substrates [2]. The other oxidases tend to oxidize specific substrates such as ascorbate, bilirubin, iron, copper, or manganese [1].

Most enzymes in the MCO family contain four copper atoms, which are essential for oxidase activity. The T1 copper has a strong absorption at ∼600 nm, which is responsible for the deep blue color of these proteins [2], [3]. The T2 copper is almost undetectable in the visible region [2]. The T3 copper site contains two coupled coppers, and shows an absorption maximum at ∼330 nm [3]. The four coppers form two copper centers: the T1 center consists of one T1 copper, and the trinuclear center consists of one T2 copper and the pair of T3 coppers [4]. A typical MCO contains three contiguous cupredoxin-like domains [1], [5]. The T1 center is located in cupredoxin-like domain 3, and the trinuclear center is located at the interface of domains 1 and 3 [5]. The four copper atoms are coordinated by ten conserved histidines (His) and one conserved cysteine (Cys) [1]. During an oxidation event, one electron is transferred from the substrate to the T1 center, and then the electron is passed via intramolecular residues (a conserved His-Cys-His triad) to the pair of T3 copper ions [4]. Molecular oxygen bound between the T3 copper ions is reduced to water after the transfer of four electrons [4].

We have been interested in insect MCOs because they function in essential physiological processes such as cuticle sclerotization and iron metabolism, and because they are potential targets for chemical control of insects [6]–[9]. During our studies of insect MCOs, we noticed that the *Tribolium castaneum* (red flour beetle) and *Anopheles gambiae* (African malaria mosquito) genomes contained an unusual MCO-like gene, which we named multicopper oxidase-related protein (MCORP). The MCORP sequences are similar to MCO sequences, but they lack the conserved cysteine and many of the conserved histidines that coordinate copper ions. Given their lack of conserved copper binding residues, it seemed unlikely that the MCORPs function as oxidases; however, their presence in two divergent insect species suggested that they may have an important physiological function. The goal of this study was to investigate the biochemical features and possible physiological functions of MCORP. To accomplish these goals, we performed phylogenetic and sequence analyses of putative insect MCORPs, purified recombinant *T. castaneum* MCORP (TcMCORP) and evaluated its copper content and oxidase activity, determined expression profiles of TcMCORP and *A. gambiae* MCORP (AgMCORP), and analyzed loss-of-function phenotypes. We found that although MCORP had no detectable oxidase activity, it is essential for viability and reproduction.

## Materials and Methods

### cDNA Cloning

To obtain a cDNA clone of TcMCORP, primers were designed based on a predicted sequence (NCBI accession number: XP_967121). To exclude the predicted signal anchor, the first 30 codons of TcMCORP were omitted from the cDNA. A cDNA pool from *T. castaneum* pupae was used as the template. To obtain cDNA clones of AgMCORP, Rapid Amplification of cDNA Ends (RACE) method was used. Primers for 5′ and 3′ ends were designed based on an EST consensus sequence (GenBank # BX611297), and 5′ and 3′ regions were cloned with a RACE kit (GeneRacer, Invitrogen). A cDNA pool from *A. gambiae* pupae was used as the template. Using primers corresponding to 5′ and 3′ untranslated sequences, a cDNA containing the full coding region of AgMCORP was amplified. All primers are listed in Table S1. Nucleotide sequences of TcMCORP and AgMCORP were confirmed by DNA sequencing (NCBI accession number: TcMCORP, KJ500311; AgMCORP, KJ500312).

### Phylogenetic and Sequence Analysis

Insect MCORP sequences were identified by performing BLAST searches of the National Center for Biotechnology Information (NCBI) non-redundant protein database using the AgMCORP sequence as the query. In addition, we did a BLAST search of the *Manduca sexta* genome, which was sequenced by the Baylor College of Medicine Human Genome Sequencing center (www.hgsc.bcm.edu) and is available at the Agricultural Pest Genomics Resource Database (www.agripestbase.org). To identify any putative MCORP orthologs in vertebrates, a BLAST search against the NCBI non-redundant protein database limited to vertebrata was performed. To identify any putative MCORP orthologs in non-insect invertebrates, a BLAST search against the NCBI non-redundant protein database excluding sequences from vertebrates, insects, fungi and plants was performed. Phylogenetic analysis was performed using MEGA5 software [10]. The highly variable amino-terminal and carboxyl-terminal ends of the MCORP and MCO sequences were not included in the alignment. Sequences beginning with the cysteine-rich region [11] were aligned by ClustalW in MEGA5 and manually adjusted (Figure S1). Gaps were omitted from the phylogenetic analysis. The phylogenetic tree was constructed by the neighbor-joining method with a Poisson model. Statistical analysis was performed by the bootstrap method with 1000 repetitions. The sequences and their NCBI accession numbers are listed in Table S2. To identify the presence or absence of putative copper-coordinating residues in MCORP sequences, the sequences of Fet3p (NCBI accession number: NP_013774) and each MCORP were aligned by ClustalW2 (EMBL-EBI), and the conserved residues coordinating four coppers in Fet3p were compared with the corresponding residues in MCORPs. Signal anchors were predicted by SignalP 2.0 Server [12]. Subcellular localization was predicted with PSORT II [13].

### Production of Polyclonal Antiserum

The cDNA of TcMCORP, beginning with the codon for P31, and the cDNA of AgMCORP, beginning with the codon for V26, were cloned into the *E. coli* expression vector, pET-28a (Novagen). The truncated fusion proteins were expressed in MAX Efficiency DH5α Competent Cells (Invitrogen), and purified by nickel affinity chromatography under denaturing conditions. Purified MCORPs were subjected to SDS-PAGE. The gels were stained with E-Zinc Reversible Stain Kit (Thermo Scientific), and the protein bands were excised and sent to Open Biosystems for the production of polyclonal antiserum in rabbits. Antisera were used at a 1∶2000 dilution for immunoblot analysis of TcMCORP and 1∶1000 dilution for immunoblot analysis of AgMCORP.

### Expression and Analysis of Recombinant AgMCORP

A secreted form of AgMCORP was expressed using a *Drosophila* S2 cell expression system (Invitrogen). The cDNA (NCBI accession number: KJ500312) beginning with the codon for A39 (without a predicted N-terminal signal anchor and several hydrophobic residues) was cloned into pMT/Bip/V5-His A (Invitrogen), which encodes a *D. melanogaster* Bip signal peptide. Primers are listed in Table S1. *D. melanogaster* S2 cells (4×10^6^ cells/mL) were transfected with recombinant DNA together with pCoBlast using calcium phosphate, and the cells were selected in Schneider’s *Drosophila* medium + 10% fetal bovine serum containing 25 µg/mL of blasticidin for two weeks. After selection, expression of AgMCORP was induced by 0.5 mM copper sulfate. After three days of expression, the stable cells were removed by centrifugation (500×*g* for 15 min). To test whether AgMCORP aggregated in the medium, 10 mL medium was concentrated to 0.5 mL with the use of Amicon Ultracel 30 K centrifugal filter. Then 10 mL buffer (20 mM MES+150 mM NaCl, pH 6.5) was added, and the sample was concentrated to ∼1 mL. The sample was analyzed by chromatography using a Superdex 200 10/300 GL column (GE Healthcare Life Sciences). After the gel filtration, immunoblot analysis was used to detect the fractions containing AgMCORP.

Full length AgMCORP was first expressed using the *Drosophila* S2 cell expression system (Invitrogen). The full length AgMCORP cDNA was cloned into pMT/V5-His A vector (Invitrogen). Primers are listed in Table S1. After selection of stable cell lines, the protein expression was extremely low. To improve expression of the full length AgMCORP, we switched to the Bac-to-Bac baculovirus expression system (Invitrogen). The full-length AgMCORP cDNA was cloned into pFastBac1, and after the DNA sequence was confirmed to be correct, a recombinant baculovirus was generated. Plaque assays were used to determine titers of amplified virus stocks. For expression, Sf9 cells (2×10^6^ cells/mL in SF900 II serum free medium supplemented with 0.1 mM copper sulfate) were infected with baculovirus at a multiplicity of infection of 2, and cells were incubated at 27°C with shaking at 140 rpm for 24 h. After incubation, cells were pelleted by centrifugation (250×*g* for 5 min).

Membrane bound proteins from Sf9 cells infected with recombinant AgMCORP baculovirus were extracted using a DUALXtract total membrane protein extraction kit (Dualsystems Biotech), with minor modifications. Briefly, cells were washed with ice-cold Cell Wash Solution and permeabilized by ice-cold Cell Permeabilization Buffer (brief vortex and continuous rocking for 10 min at 4°C). Cells were pelleted by centrifugation (16000×*g* for 15 min) at 4°C. The supernatant was saved as a cytoplasmic protein fraction. Ice-cold Membrane Protein Extraction Buffer (containing CHAPS) was added to cell pellets. The cells and buffer were mixed by pipetting. The mixture was incubated for 30 min at 4°C with vigorous shaking, and then was centrifuged at 16000×*g* for 15 min. Both the supernatant (extractable membrane protein fraction) and the final cell debris (including unextracted membrane proteins) were saved.

In addition, we used another method to extract membrane proteins without the use of Cell Permeabilization Buffer, and, in addition to CHAPS, we tested another mild detergent, octyl-β-glucoside. Briefly, cell pellets were resuspended in buffer (20 mM MES+150 mM NaCl, pH 6.5). The sample was sonicated using 30 sec bursts, 4 times. Then, ultracentrifugation (100000×*g* for 60 min) was performed to pellet membranes, membrane proteins and cell debris. The supernatant was saved as a cytoplasmic protein fraction. The membrane proteins were extracted using CHAPS or 100 mM octyl-β-glucoside. The cells and buffer were mixed by pipetting. The cells were incubated with CHAPS for 1 h or octyl-β-glucoside for 30 min at 4°C with vigorous shaking, and then were centrifuged at 16000×*g* for 15 min. Both the supernatant (extractable membrane protein fraction) and the cell debris (including unextracted membrane proteins) were saved.

### Expression and Purification of Recombinant TcMCORP

TcMCORP was expressed using a baculovirus expression system (flashBAC, Oxford Expression Technologies). A cDNA containing the coding region of TcMCORP without a predicted N-terminal signal anchor was first cloned into pMT/BiP/V5-His A vector (Invitrogen), which encodes a *D. melanogaster* Bip signal peptide. The signal peptide-TcMCORP fusion cDNA was then cloned into the pOET3 baculovirus transfer vector. Primers are listed in Table S1. Sf9 cells were transfected, and recombinant baculovirus was generated. Plaque assays were performed to determine titers of amplified virus stocks. For expression, 2.8 L of Sf9 cells (2×10^6^ cells/mL) were infected with baculovirus at a multiplicity of infection of 1, and cells were incubated at 27°C with shaking at 140 rpm for 24 h. SF900 II serum free medium was used and supplemented with 0.1 mM copper sulfate to provide copper to the recombinant protein. After one day of expression, cells were removed by centrifugation (500×*g* for 15 min).

For purification, MCORP and other glycosylated proteins in the cell culture medium were bound to concanavalin-A-Sepharose and eluted with 0.5 M methyl-α-D-mannopyranoside in 20 mM Tris-HCl, 0.5 M NaCl, pH 7.4 (4°C). Then, eluted proteins were dialyzed against 50 mM sodium phosphate, pH 6.5 (4°C) and loaded on a HiPrep SP FF 16/10 column (GE Healthcare Life Sciences). Proteins were eluted with a linear gradient of NaCl (0–1 M) in 50 mM sodium phosphate, pH 6.5. Fractions containing TcMCORP were pooled and concentrated to 5 mL with the use of an Amicon Ultracel 30 K centrifugal filter. The final purification step was accomplished by a Superdex 200 HiLoad 16/60 column (GE Healthcare Life Sciences) using 50 mM sodium phosphate, 150 mM NaCl, pH 6.5. At each purification step, immunoblot analysis was used to detect the fractions containing TcMCORP. The concentration of purified recombinant TcMCORP was estimated as described previously [14]. The yield was ∼1 mg per liter of cell culture. The purified protein was subjected to SDS-PAGE and analyzed by immunoblot. Purified TcMCORP was stored at 4°C.

### Spectral Properties and Inductively Coupled Plasma Mass Spectrometry (ICP-MS)

For spectral properties, purified recombinant TcMCORP was concentrated to 4 µg/µL with the use of an Amicon Ultracel 30 K centrifugal filter. The concentration was estimated as described previously [14]. AgMCO3 [14] (4 µg/µL) was used as a positive control. Absorption spectra of TcMCORP and AgMCO3 were recorded on a Beckman DU-640 Spectrophotometer. Absorption was read from 250 to 700 nm at 1 nm intervals.

For ICP-MS analysis, TcMCORP in 50 mM sodium phosphate, 150 mM NaCl, pH 6.5, was concentrated to 8 µg/µL (0.1333 mM). TcMCORP and the storage buffer were diluted 12-fold immediately prior to manual analysis through a peristaltic pump at 0.4 mL/min serving a Babbington nebulizer and cooled (2°C) spray chamber serving a standard quartz torch and 1400 W argon plasma. Data were recorded in 5 second replicates and manually processed for the small volume received. They were calibrated to NIST-traceable standards which are validated with NIST standard reference material solutions. The metal content of TcMCORP was calculated as the metal content of the TcMCORP sample minus the metal content of the storage buffer.

### Activity Assays

The laccase substrates used were catechol, hydroquinone, methyl hydroquinone, L-dopa, dopamine, *N*-acetyldopamine (NADA), *N*-β-alanyldopamine (NBAD), *o*-phenylenediamine, guaiacol, 2-aminophenol, 2,2′-azino-bis(3-ethylbenzothiazoline-6-sulphonic acid) (ABTS), syringaldazine, and *N,N*-dimethyl-*p*-phenylenediamine. Reactions to determine enzyme activity were made by mixing 5 µg purified recombinant TcMCORP with 1 mM substrate (except for syringaldazine, which was used at 10 µM because of its low solubility in the assay buffer) in a total volume of 200 µL and observing the change in absorbance for 30 min to detect product formation using a microplate spectrophotometer. All assays were done in duplicate in citrate-phosphate buffer at pH 5, 6, or 7. Reactions with no TcMCORP were done to measure autooxidation of substrates and served as “blank” reactions. Wavelengths used for detecting the products of interest were: 450 nm for catechol, 250 nm for hydroquinone and methyl hydroquinone, 475 nm for L-dopa and dopamine, 390 nm for NADA and NBAD, 440 nm for *o*-phenylenediamine, 436 nm for guaiacol, 434 nm for 2-aminophenol, 414 nm for ABTS, 530 nm for syringaldazine, and 515 nm for *N,N*-dimethyl-*p*-phenylenediamine.

### RNA Isolation, cDNA Synthesis and RT-PCR

For a developmental expression profile of TcMCORP, *T. castaneum* at different developmental stages (eggs, younger larvae, older larvae, pharate pupae, pupae, and adults) were collected for RNA isolation. For a tissue expression profile of TcMCORP, ovaries, guts and Malpighian tubules were removed from *T. castaneum* females (adult Day 6), and the remainder of the abdomen (“carcass”) was saved. For a developmental expression profile of AgMCORP, *A. gambiae* at different developmental stages (eggs from an overnight collection, older eggs that were between one and two days old (and, thus, included mostly pharate larvae), 1^st^–4^th^ instar larvae, pupae, pharate adults, and adult females) were collected for RNA isolation. For a tissue expression profile of AgMCORP, tissues from *A. gambiae* adult females and males were dissected as described previously [15]. For all expression profiles, pools of at least 10 individuals were used for each sample. RNA isolation and cDNA synthesis were done as described previously [8]. 1 µL of cDNA was used for each RT-PCR. All primers are listed in Table S1.

### Insect Culture

For mosquito culture, the G3 strain of *A. gambiae* was obtained from the Malaria Research and Reference Reagent Resource Center. The mosquitoes were reared as described previously [15]. For *T. castaneum* culture, the GA-1 strain was reared in whole wheat flour containing 5% brewer’s yeast at 30°C under standard conditions [16].

### RNAi to Determine Loss-of-function Phenotypes

TcMCORP, *T. castaneum* vermillion (TcVer), AgMCORP, and GFP dsRNAs were synthesized by using the MEGAscript RNAi kit (Ambion). TcVer [17] and GFP served as negative controls. Primers are listed in Table S1. dsRNAs were injected by using a microinjection system (Nanoliter 2000 and Micro4 controller) and a stereomicroscope. To evaluate the dsRNAs for possible off-target effects, we used E-RNAi [18]. The software identified no off-targets of dsAgMCORP. E-RNAi predicted that the TcMCORP dsRNA would generate 358 possible 19-nucleotide matches to TcMCORP, one possible 19-nucleotide match to Tc10662, and one possible 19-nucleotide match to Tc005584. No matches of ≥20 nucleotides were found.

*T. castaneum* late larvae (larvae that have reached their maximum size – plump and long) and pharate pupae were injected with TcMCORP dsRNA or TcVer dsRNA (400 ng per insect) to assess the effects of knockdown. For adult survivorship assays, live insects were counted starting from adult day 0 (that is, the number of adults on day 0 was considered 100% survivorship). The insects were counted approximately every 3 days for one month. (The first time we did this experiment, all 49 of the dsMCORP insects were dead within 30 days, but only one of the 48 control insects had died. Because the control dsRNA (dsVer) caused no mortality, we did not repeat the dsVer injections when we performed our second and third biological replicates). For assays relevant to reproduction, females and males were separated at the pupal stage. For tissue observation, on day 10 post adult eclosion, ovaries, male reproductive tissues, guts, and Malpighian tubules were dissected from the insects and observed. For determination of egg numbers, on day 4 post adult eclosion, single pair-based crosses were performed: dsTcMCORP ♀ × wild type (WT) ♂, dsTcVer ♀ × WT ♂, dsTcMCORP ♂ × WT ♀, and dsTcVer ♂ × WT ♀. After 7 days of rearing, the egg number from each pair was determined. Pairwise differences in egg numbers were assessed by performing a nonparametric analysis (Mann-Whitney test). In order to test the efficiency of TcMCORP knockdown, total RNA was prepared from pupae (2 days post injection) with dsTcMCORP or wild type. RNA isolation and cDNA synthesis were done as described previously [8]. RT-PCR with gene specific primers was performed.

*A. gambiae* day 0 females were injected with dsRNA for AgMCORP or GFP (2 µg per insect). Injected females and WT males were mated for 7 days, and then given access to a blood meal. Females that had taken a blood meal were selected from the pool of females on day 8. On day 9, ovaries were dissected, observed, and used for testing the efficiency of knock down. Total RNA was isolated and cDNA was synthesized as described previously [8]. There were three biological replicates from dsAgMCORP and dsGFP treatments. Real-time PCR reactions were performed. Expression of AgMCORP data were normalized to RPS3 (ribosomal protein S3). Primers are listed in Table S1.

## Results

### A Gene Encoding an Unusual Multicopper Oxidase-related Protein Was Found in *T. castaneum* and *A. gambiae* Genomes

During our studies of *T. castaneum* and *A. gambiae* MCOs, we discovered that these two insect species have a gene encoding a multicopper oxidase-related protein. We used RT-PCR to clone cDNAs of these two MCORPs and analyzed their predicted amino acid sequences. The MCORP sequences are similar to MCO sequences, but many of the residues that coordinate copper ions in MCOs are absent (Figure S2). The MCORP sequences have a hydrophobic amino-terminus that resembles a signal peptide but is predicted to remain uncleaved (Figure S2). This type of amino-terminus, referred to as a signal anchor, directs a protein to the secretory pathway, but tethers the protein to the membrane so that it is not secreted [19]. Subcellular localization prediction software predicts that both MCORPs are localized to the endoplasmic reticulum. The sequences also contain a cysteine-rich region that is present in all known insect MCOs [11], and three putative cupredoxin-like domains (Figure S2).

### MCORP Orthologs Belong to a Distinct Clade

**Figure 1:**
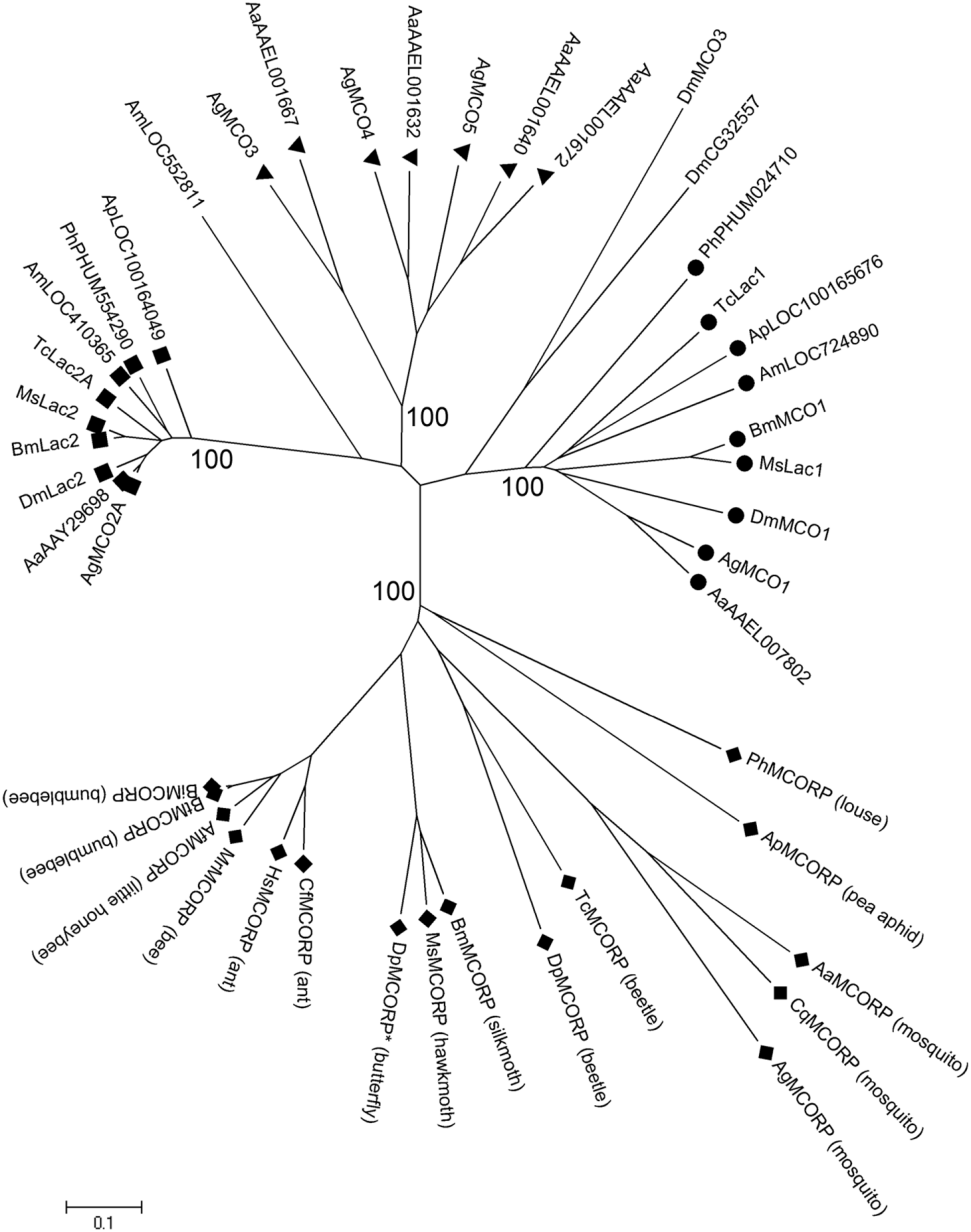
Phylogenetic tree of insect MCOs and MCORPs. The tree was constructed by the neighbor-joining method. Numbers shown are bootstrap values expressed as a percentage. Bootstrap values are only shown for the MCORP branch (diamond), MCO1 branch (circle), MCO2 branch (square), and the mosquito-specific branch (triangle). All identified MCORPs were included in the analysis, and MCOs from insect species that represent different insect orders were included, Abbreviations used are: Ag, *Anopheles gambiae*; Aa, *Aedes aegypti*; Cq, *Culex quinquefasciatus*; Tc, *Tribolium castaneum*; Dp, *Dendroctonus ponderosae*; Ms, *Manduca sexta*; Bm, *Bombyx mori*; Dp*, *Danaus plexippus*; Cf, *Camponotus floridanus*; Hs, *Harpegnathos saltator*; Bt, *Bombus terrestris*; Bi, *Bombus impatiens*; Mr, *Megachile rotundata*; Af, *Apis florae*; Ap, *Acyrthosiphon pisum*; Ph, *Pediculus humanus corporis*; Dm, *Drosophila melanogaster*; Am, *Apis mellifera*. Accession numbers are listed in Table S2.

To determine whether MCORP is a conserved insect gene, we used BLAST to search for similar sequences in the NCBI non-redundant protein database. We identified putative MCORP orthologs in six orders of insects: Diptera, Coleoptera, Lepidoptera, Hymenoptera, Hemiptera, and Anoplura. MCORP orthologs were identified in three dipteran species (all mosquitoes), but not in *Drosophila* species. We did not find MCORP orthologs in other invertebrates or vertebrates. A phylogenetic analysis of MCORPs and insect MCOs clearly shows that MCORPs belong to a specific cluster with a bootstrap value of 100 (Figure 1). Thus, MCORP is present in a diverse set of insect species, suggesting that MCORPs may have a conserved function.

### MCORPs Lack Many of the Conserved Copper-coordinating Residues in MCOs

**Figure 2:**
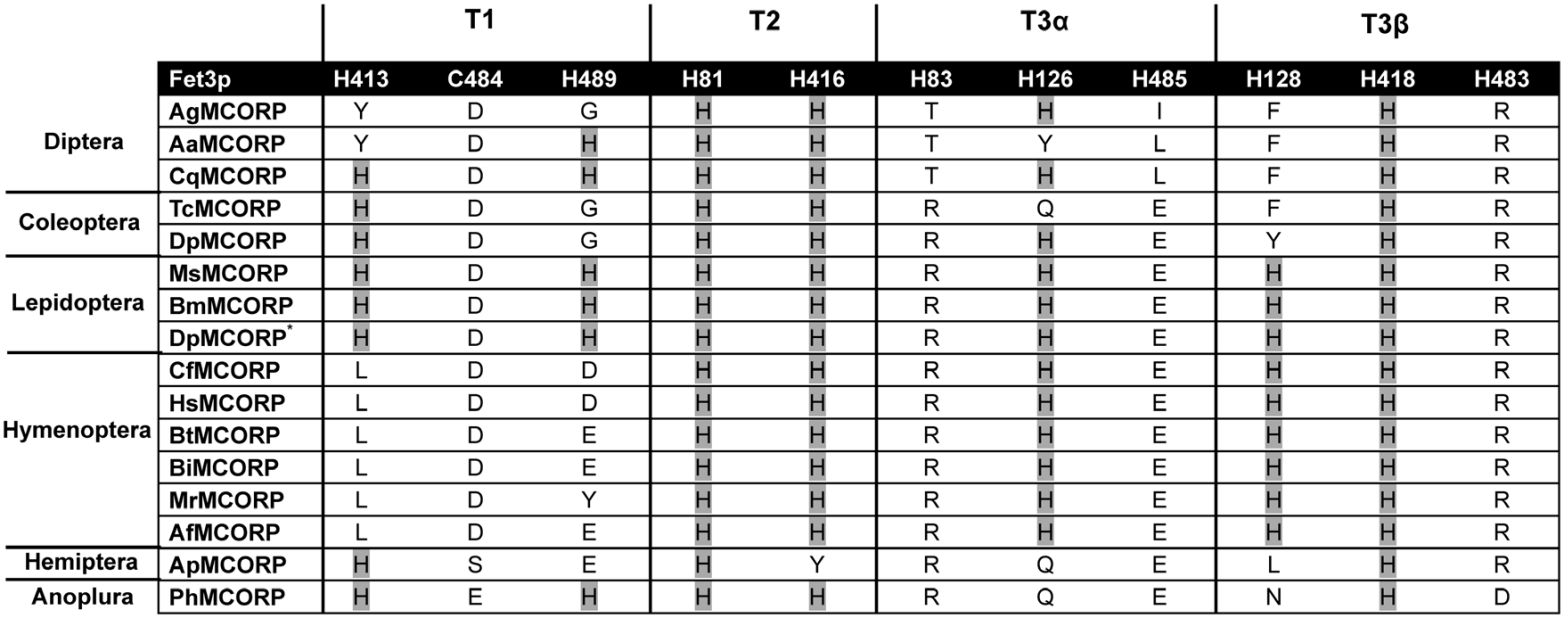
Comparison of copper-coordinating residues in Fet3p with the corresponding residues in MCORPs. Yeast multicopper ferroxidase, Fet3p, contains 10 histidines and 1 cysteine that coordinate to four coppers. T1 copper is coordinated with H413, H489 and C484, T2 copper is coordinated with H81 and H416, T3α copper is coordinated with H128, H418 and H483, and T3β copper is coordinated with H83, H126 and H485. Fet3p was aligned with each MCORP, and the corresponding residues in MCORPs are shown. Histidines conserved in MCORPs are highlighted in gray. Abbreviations used are the same as for Figure 1.

Almost all MCOs have ten conserved histidines and one conserved cysteine that form two copper centers. Each MCORP ortholog was aligned with a well-studied yeast multicopper ferroxidase, Fet3p, whose structure is solved [20]. The conserved copper-coordinating residues in Fet3p and the corresponding residues in MCORPs were analyzed (Figure 2). The MCORP sequences contain two histidines that align with the T2 copper coordinating residues in typical MCOs; however, most of the other copper-coordinating residues are missing. In MCOs, an electron is transferred from the T1 copper to T3 coppers through a highly conserved tripeptide (His-Cys-His) comprising three of the copper coordinating residues [2]. For Fet3p, the tripeptide is H485, C484, and H483 [4]. Notably, these three residues are missing in MCORPs (Figure 2). Based on our sequence analysis, we conclude that if any electron transfer occurs in MCORPs, it must happen differently from the electron transfer in MCOs.

### Recombinant AgMCORP Is Membrane-bound

**Figure 3:**
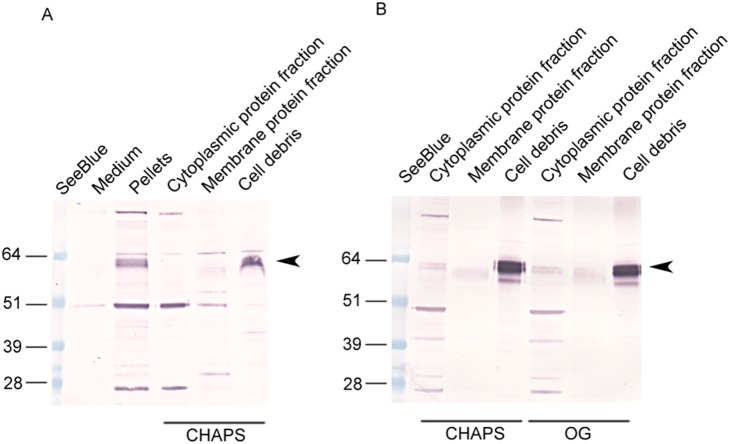
Expression and immunoblot analysis of recombinant full length AgMCORP. A) Membrane proteins were extracted using DUALXtract total membrane protein extraction kit (Dualsystems Biotech). After 24****h expression, cells were pelleted by centrifugation. Both the supernatant and pellets were analyzed. Full length AgMCORP (66****kDa) was not detected in the medium, but was present in pellets. After extraction by CHAPS, AgMCORP was not in the cytoplasmic protein fraction or extractable membrane protein fraction, but in the cell debris. B) Membrane proteins were extracted using a different procedure. The procedure included sonication and ultracentrifugation to separate cytoplasmic proteins and membrane proteins. Two mild detergents, CHAPS and OG (octyl-β-gluside), were used to extract the membrane fraction. AgMCORP was not extracted by the detergents and remained in the cell debris fraction.

In order to determine copper content and test oxidase activity of MCORP, we wanted to express and purify recombinant MCORP using an insect cell expression system. We first tried to purify recombinant AgMCORP. To simplify protein purification, we replaced the putative signal anchor with a signal peptide so that the recombinant protein would be secreted. However, secreted recombinant AgMCORP formed aggregates and could not be purified. Then, we tried to purify full length AgMCORP. As expected, full length AgMCORP was not secreted but remained associated with the cultured cells (Figure 3A). We used mild detergents, CHAPS and octyl-β-gluciside, to extract membrane bound proteins from the cells. AgMCORP was not in the cytoplasmic protein fraction, and, it was not successfully extracted from the membrane; instead, it was present in the cell debris that contains insoluble materials, unbroken cells and lipid rafts (Figure 3). Although we failed to purify full length AgMCORP, this result suggests that AgMCORP is membrane-bound, which is consistent with the presence of an amino-terminal signal anchor.

### Recombinant TcMCORP Contains Little Copper and Does Not Have Laccase Activity

**Figure 4:**
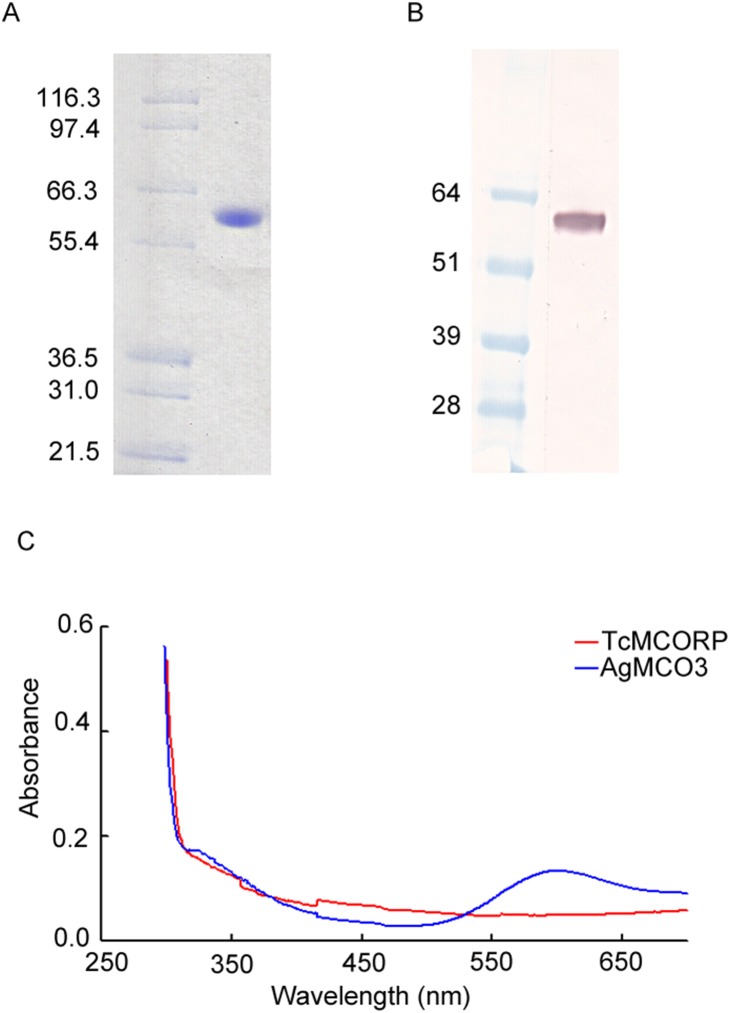
SDS-PAGE, immunoblot analysis and absorption spectrum of purified TcMCORP. A) Purity was verified by Coomassie staining. B) The identity of the purified protein was confirmed by immunoblot analysis using antiserum against TcMCORP. C) UV/vis spectra of purified TcMCORP and AgMCO3 (positive control). The concentration used was 4 µg/µL.

Unlike AgMCORP, secreted recombinant TcMCORP did not aggregate, and purification was successful. Recombinant TcMCORP was purified with the use of concanavalin-A affinity chromatography, SP cation exchange chromatography, and gel filtration chromatography. We purified 1 mg TcMCORP per liter of cell culture. SDS-PAGE followed by Coomassie staining showed that recombinant TcMCORP was approximately the expected mass (60 kDa) and was very pure (Figure 4A). The identity of TcMCORP was verified by immunoblot analysis using antiserum against TcMCORP (Figure 4B).

MCOs have a blue color due to the presence of a T1 copper [2], [3]. Consistent with our prediction that MCORP does not contain a T1 copper, we observed that highly concentrated (8 µg/µL = 0.1333 mM) recombinant TcMCORP was not blue. In addition, based on its absorption spectrum (Figure 4C), TcMCORP does not have the typical spectroscopic properties that MCOs possess. The characteristic absorption maximum at ∼600 nm and the shoulder at ∼330 nm were absent in the recombinant TcMCORP, but were present in recombinant AgMCO3 that served as a positive control (Figure 4C). Because most MCORPs contain the two conserved histidines that typically coordinate a T2 copper, we predicted that each molecule of TcMCORP may bind to one atom of copper; however, metal content analysis using inductively coupled plasma mass spectrometry (ICP-MS) demonstrated that recombinant TcMCORP contains little copper (Table 1). The ratio of copper to MCORP was one to seventeen; therefore, if each MCORP can bind to one copper, this result indicates that ∼6% of the MCORP molecules contained one copper atom. Our study cannot distinguish whether copper is bound by the two histidines that align with T2 copper-coordinating residues or whether the copper is bound elsewhere, for example, to histidines on the surface of the protein. Very small amounts of iron and zinc were also detected in the TcMCORP sample, but it seems unlikely that the presence of these metals in such low abundance is biologically relevant.

**Table 1:** ICP-MS analysis of recombinant TcMCORP.

Metal	Concentration (ng/mL)	Molarity (mM)	Molar ratio (metal:TcMCORP)
Copper	498.4	0.0078	1∶17
Zinc	165.8	0.0026	1∶51
Iron	82.1	0.0015	1∶89
Manganese	3.6	<0.0001	1∶>1000

The apparent lack of T1 and T3 coppers would suggest that TcMCORP is not an active oxidase; however, because some atypical laccases have fewer than four copper atoms [21]–[25], we decided to test whether TcMCORP has laccase activity by using a broad range of laccase substrates. In each reaction, 5 µg recombinant TcMCORP was mixed with 1 mM substrate (except for syringaldazine, which was used at 10 µM). The amount of TcMCORP was ten times more than the amount we usually use to analyze insect laccase activity, and 1 mM substrate was used because insect laccases tend to have *K_m_*s in the range of 0.1–10 mM [14], [26], [27]. Activity was tested at pH 5, 6 and 7. Predictably, no laccase activity was detected.

### MCORP Is Expressed at Each Developmental Stage and in Many Different Tissues

**Figure 5:**
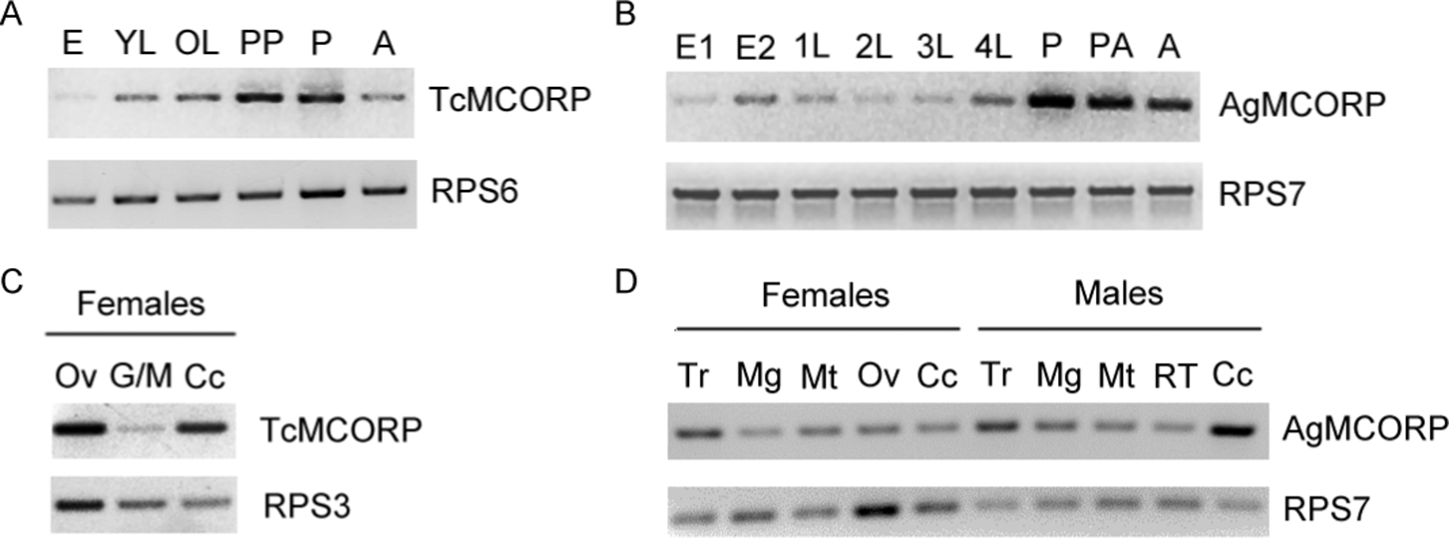
Expression profiles of MCORP in *T. castaneum* and *A. gambiae*. Results are from qualitative RT-PCR. A) Developmental expression profile of TcMCORP. Abbreviations: E, eggs; YL, younger larvae; OL, older larvae; PP, pharate pupae; P, pupae; A, adults. B) Developmental expression profile of AgMCORP. Abbreviations: E1, eggs; E2, older eggs and pharate larvae; 1L, 1^st^ instar larvae; 2L, 2^nd^ instar larvae; 3L, 3^rd^ instar larvae; 4L, 4^th^ instar larvae; P, pupae; PA, pharate adults; A, adult females. C) Tissues from *T. castaneum* adult females were analyzed. Abbreviations: Ov, ovaries; G/M, guts and Malpighian tubules; Cc, carcass. D) Tissues from *A. gambiae* adult females and males were analyzed. Abbreviations: Tr, thorax; Mg, midguts; Mt, Malpighian tubules; Ov, ovaries; RT, reproductive tissues; Cc, carcass.

To begin to learn about the biological function of MCORP, we first analyzed expression patterns of TcMCORP and AgMCORP. MCORP mRNA was detected in all developmental stages, with significant expression during metamorphosis (pharate pupal, pupal, and pharate adult stages) (Figure 5A and B). The high number of PCR cycles (>30) required to generate detectable PCR products suggest that MCORP is expressed at a relatively low level. In order to investigate the tissues in which MCORP is expressed, RNA was isolated from various adult tissues followed by RT-PCR. TcMCORP mRNA was detected in all of the tissues analyzed, including ovaries (Figure 5C). Similarly, AgMCORP mRNA was detected in midguts, Malpighian tubules, ovaries, male reproductive tissues, abdominal carcass (mainly fat body), and thorax (Figure 5D). In addition, AgMCORP transcripts have been detected by microarray studies in midguts, fat bodies, ovaries, heads, and hemocytes [28], [29]. Taken together, the expression data indicate that TcMCORP and AgMCORP are constitutively expressed at a low level in many different tissues.

### Knockdown of TcMCORP Results in Mortality

**Figure 6:**
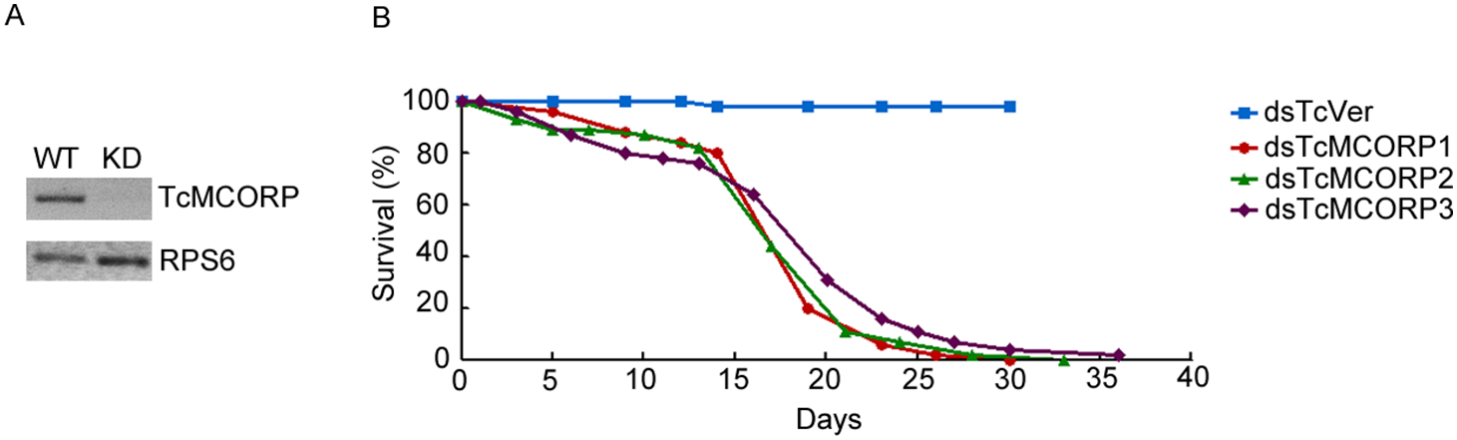
Efficiency of TcMCORP knockdown and effect of TcMCORP knockdown on adult life span. A) Whole bodies from WT *T. castaneum* pupae and from pupae with dsTcMCORP treatment (KD) 2 days post injection were analyzed. B) *T. castaneum* pharate pupae were injected with TcMCORP dsRNA or TcVer dsRNA. Live insects were counted starting from adult day 0. The life span of TcMCORP knockdown beetles was shorter than that of control beetles (TcVer knockdown). For TcMCORP knockdown, data are from three biological replicates (n = 49, 45, or 55). For TcVer knockdown data are from one biological replicate (n = 48).

To detect MCORP loss-of-function phenotypes in *T. castaneum*, we used RNAi-mediated knockdown. Insects were injected with dsRNA that targets TcMCORP or TcVer. The insects injected with dsTcVer served as negative controls. Transcripts for TcMCORP were suppressed efficiently two days after dsRNA injections (Figure 6A). When late larvae were injected with dsTcMCORP, insects died at either the pharate pupal stage (did not molt to pupae) or late pharate adult stage (did not molt to adults), and none of them survived to the adult stage. These results indicate that TcMCORP is an essential gene. When pharate pupae were injected with dsTcMCORP, approximately 20% of insects died during the pupal to adult transition (compared with only ∼2% mortality following dsTcVer treatment). Among the dead insects, some died very late in the pharate adult stage (just before eclosion), and some died at the time of adult eclosion, with incomplete shedding of the pupal cuticle. Successful eclosion occurred in the rest of the treated insects, but the dsTcMCORP-injected insects had a much shorter adult life span than control insects (Figure 6B). Compared to the controls, the number of dead dsTcMCORP insects increased gradually in the first two weeks of adult life, and then increased drastically, resulting in almost 0% survivorship one month after eclosion. The external morphology of dead adults in the dsTcMCORP treatment group was similar to that of live adults in the dsTcVer treatment group. The midguts dissected from live dsTcMCORP-injected adults contained food, which indicates that the insects could eat when they were alive and suggests that the dsTcMCORP insects did not starve.

### Knockdown of TcMCORP in Females Reduces the Number of Eggs Laid

**Figure 7:**
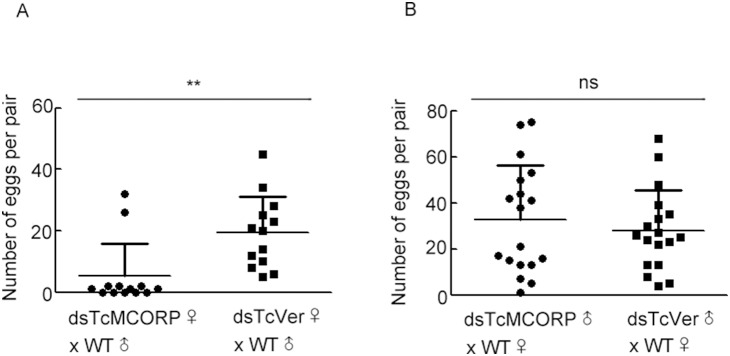
Effect of TcMCORP knockdown on egg number. dsRNA targeting TcMCORP or TcVer was injected into *T. castaneum* pharate pupae. Injected females and males were sexed at the pupal stage. At 4 days after adult eclosion, the RNAi insects were mated with wild type (WT) insects on a single pair basis. The eggs produced from each pair were counted. Pairwise differences in egg numbers were assessed by performing a Mann-Whitney test. Bars represent mean + standard deviation. A) Comparison of the number of eggs produced from dsTcMCORP ♀ × WT ♂ crosses and dsTcVer ♀ × WT ♂ crosses. The latter group served as a control. A significant difference was observed (** = p<0.01, n = 13). B) Comparison of the number of eggs produced from dsTcMCORP ♂ × WT ♀ crosses and dsTcVer × WT ♀ crosses. The latter group served as a control (ns = not significant, n = 18).

We observed that dsTcMCORP-treated adults had very few progeny; therefore, we were interested in whether knockdown of MCORP had an effect on reproductive processes. A single pair-based cross design was used to evaluate egg numbers from knockdown females mated to control males and from knockdown males mated to control females. The number of eggs produced from dsTcMCORP ♀ × wild type (WT) ♂ crosses was much less than the number from control crosses (Figure 7A). Eleven out of thirteen pairs of dsTcMCORP ♀ × WT ♂ produced 0–2 eggs, although two pairs produced more. The outliers might be the result of poor knockdown efficiency in those females or the result of phenotypic variability. The number of eggs produced from dsTcMCORP ♂ × WT ♀ crosses was similar to that from control crosses (Figure 7B) and no obvious difference in egg hatching was observed. Thus, RNAi of TcMCORP in females caused a significant reduction in egg production, but knockdown in males had no observable effect on reproduction.

### Knockdown of TcMCORP Inhibits Oocyte Maturation

**Figure 8:**
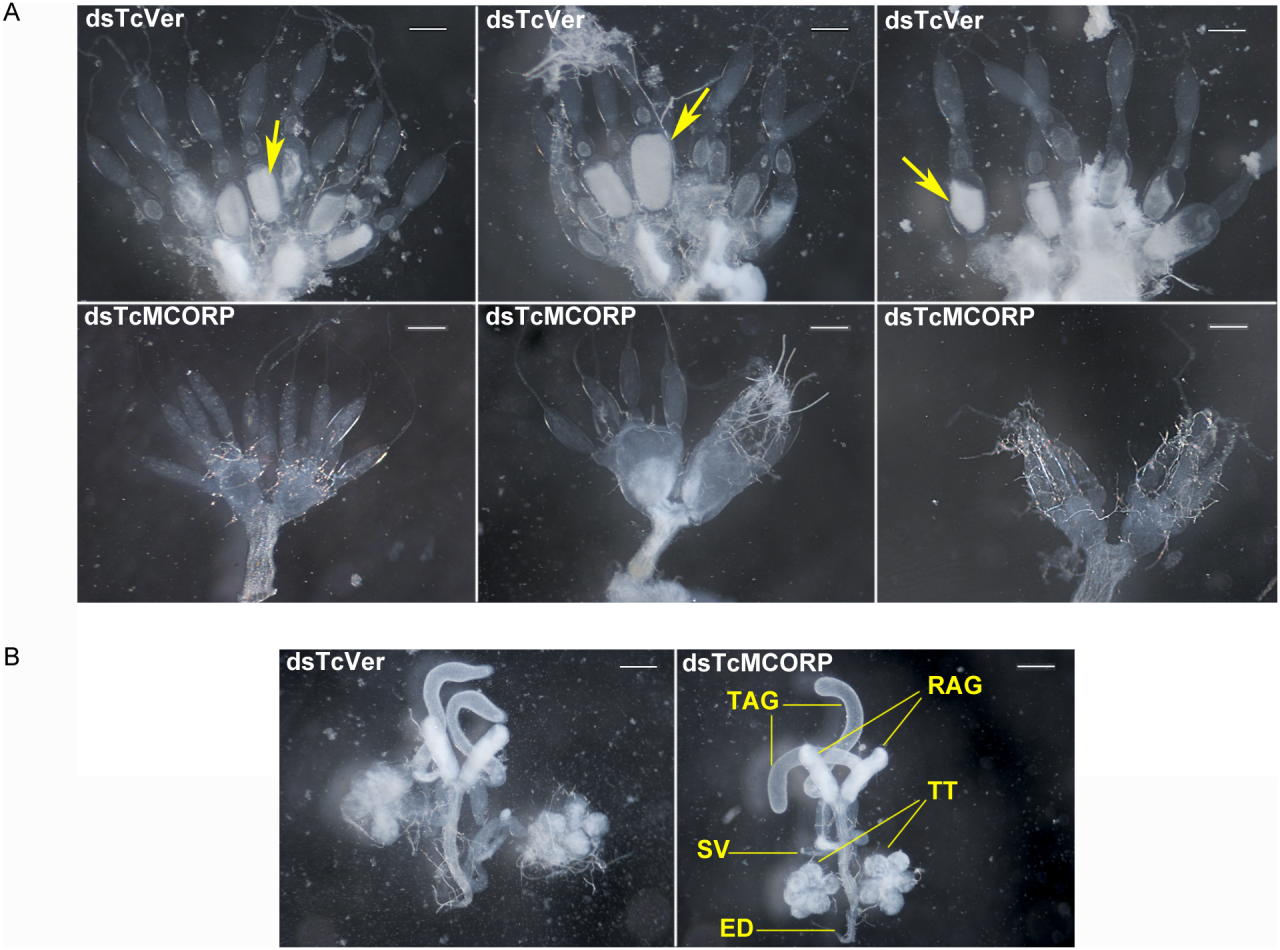
Effect of RNAi on the reproductive tissues in *T. castaneum*. A) The top three images represent ovaries dissected from TcVer knockdown insects (control), and the bottom three images show ovaries dissected from TcMCORP knockdown insects. In each image, the ovary is oriented with nurse cells on top and oocytes at the bottom. The mature primary oocytes are marked by a yellow arrow. Note that primary oocyte development was blocked in TcMCORP RNAi insects but not in TcVer RNAi insects. For dsTcVer treatment, all ovaries (n = 12) were fully developed. For dsTcMCORP treatment, 14 out of 18 insects showed the absence of mature primary oocytes. Scale bar: 200 µm. B) The left image shows the male reproductive tissue dissected from a TcVer knockdown insects (control), and the right image shows the male reproductive tissue from a TcMCORP knockdown insect. All male reproductive tissues dissected from TcMCORP knockdown insects (n = 15) and TcVer controls (n = 8) looked normal. Abbreviations: TAG, tubular accessory glands; RAG, rod-shaped accessory glands; SV, seminal vesicle; TT, testes; ED, ejaculatory duct. Scale bar: 200 µm.

A decrease in egg production by females treated with dsTcMCORP suggested that MCORP may be required for ovary development. To test this hypothesis, we assessed the effect of TcMCORP knockdown on ovary morphology. *T. castaneum* pharate pupae were injected with dsRNA, and females and males were separated at the pupal stage so that no mating would occur. Ten days after adult eclosion, the insects were dissected, and ovaries, male reproductive tissues, guts, Malpighian tubules and fat bodies were observed. Knockdown of TcMCORP expression seriously impaired the maturation of the primary oocytes, whereas TcVer RNAi had no effect (Figure 8A). TcMCORP knockdown had no visible effect on the reproductive tissues in males (Figure 8B). These results are consistent with our observation that knockdown of TcMCORP in females greatly reduced egg production and that knockdown in males had no effect. In addition, there were no obvious differences in other tissues from dsTcMCORP and dsTcVer treatments; therefore, the mortality observed in dsTcMCORP-treated adults was not associated with any obvious morphological changes in these tissues.

We wondered whether knockdown of MCORP would also affect ovary development in *A. gambiae*. Injection of larvae or pupae leads to high mortality in *A. gambiae*; therefore, we injected day 0 females with dsRNA that targets AgMCORP or GFP (negative control). Injected females and WT males were mated and then given a blood meal, which triggers oocyte development in anopheline mosquitoes [30]. Mosquitoes subjected to AgMCORP RNAi had fully developed ovaries, similar to those subjected to GFP RNAi. One interpretation of this result is that MCORP is not required for ovary development in *A. gambiae*; however, the negative result may be due to one of two technical difficulties: incomplete knockdown (65% based on qPCR), despite injecting the maximum amount of dsRNA per insect (2 µg), or dsRNA injection in the adult stage, after the ovaries were already partly developed. More sophisticated RNAi methods, which are currently unavailable, will be necessary to determine whether MCORP is required for oocyte development (or viability) in mosquitoes.

## Discussion

This study describes a novel protein, MCORP, which shares sequence similarity with MCOs, but lacks most of the highly conserved residues necessary to coordinate the T1 and T3 copper atoms. Without the T1 and trinuclear copper centers, it seemed unlikely that MCORP would be catalytically active; however, there have been several studies of atypical fungal laccases that contain fewer than four copper atoms [21]–[25]. These laccases were termed “white” laccases because they lack the blue color of most MCOs. Those studies focused on the biotechnological applications of the unusual laccases rather than their biochemical features; therefore, the amino acid sequences, including the presence or absence of conserved copper-coordinating residues, were not reported. We found that, unlike the atypical laccases, recombinant TcMCORP had no detectable laccase activity. MCORP may oxidize a natural substrate that we did not test, but we think this is unlikely because lacccases oxidize a broad range of substrates, and even MCOs with preferences for non-phenolic substrates (such as iron or ascorbate) can oxidize laccase substrates [8], [31], [32].

Whether MCORP is a copper-binding protein is still unclear. Because most MCORPs have two conserved histidines that correspond to the T2 copper-coordinating residues in typical MCOs, we thought that MCORPs may bind one atom of copper. However, we found that recombinant TcMCORP contains very little copper. The low copper content could be the result of unknown technical problems with recombinant protein expression, although we have used the same insect cell culture system to produce several active insect MCOs [8], [14], [26], [27]. If the copper content of recombinant TcMCORP reflects the copper content of endogenous TcMCORP, our results might suggest that TcMCORP is a copper-binding protein that does not require copper as a cofactor. For example, if TcMCORP participates in copper sensing or copper transport, we would expect only some TcMCORP molecules to contain copper. A third possibility is that endogenous MCORP does not bind copper, and the small amount of copper present in recombinant MCORP may be due to binding of copper to the surface of MCORP due to the high concentration of copper in the cell culture medium.

MCORPs have not been described previously, but an analogous type of protein has been identified in plants. These proteins are similar to ascorbate oxidases, but, like the insect MCORPs, lack copper-coordinating residues [33]. The best-studied of these ascorbate oxidase homologs, SKU5 from *Arabidopsis thaliana*, shares 23% sequence identity with ascorbate oxidases. The residues required for coordinating the T1 and T3 copper ions are absent in SKU5, but the two histidines required for coordinating the T2 copper are present. SKU is expressed in all tissues, most strongly in expanding tissues, and it is involved in directional root growth [34]. Bp 10 from *Brassica napus* shares 30% sequence identity with ascorbate oxidases, lacks most of the conserved copper-coordinating ligands, and is expressed in developing pollen [35]. Whether SKU5 and Bp10 bind to copper and whether they have oxidase activity are unknown. These ascorbate oxidase homologs are more closely related to ascorbate oxidases in plants than to insect MCOs [33].

In our study, knockdown of TcMCORP in females inhibited oocyte maturation and, thus, egg production. Female reproduction in insects includes vitellogenesis and oogenesis. During vitellogenesis, yolk proteins, such as vitelloginin (Vg), are synthesized in the fat body, secreted into the hemolymph and accumulate inside the oocyte. In addition to vitellogenesis, successful reproduction requires ovarian growth and oocyte maturation. Insect reproduction is regulated by complex molecular networks. For example, insect Vg and lipophorin (Lp) receptors play an important role in oocyte development by mediating endocytosis of Vg and Lp [36]; hormones such as juvenile hormone (JH) and 20-hydroxyecdysone (20E) regulate insect reproduction [37]. In *T. castaneum*, female reproduction is regulated by JH, 20E and nutritional signaling [37]. JH regulates Vg synthesis in the fat body [38], 20E regulates oocyte maturation [39], and nutritional signals play key roles in both Vg synthesis and oocyte maturation [37]. In addition to the studied hormones and nutritional signals, other genes might also regulate Vg synthesis and/or oocyte maturation [38], [39]. Currently, we do not know the molecular mechanisms of TcMCORP involvement in insect reproduction, but our study adds new knowledge to this topic.

We found that TcMCORP larval RNAi led to 100% mortality prior to adult eclosion. These deaths occurred during the pharate pupal stage or late pharate adult stage. Pharate pupal RNAi resulted in 20% mortality during the pupal to adult transition, and 100% mortality by one month after adult eclosion. We have been unable to determine the cause of the deaths, but they do not seem to be due to starvation or gross morphological abnormalities. Given the constitutive, ubiquitous expression of MCORP and its lethal knockdown phenotype, we hypothesize that MCORP may play a role in a basic cellular process that is required during the transition from larval to pupal stage and pupal to adult stage. Because MCORP function is essential for insect viability and reproduction and because MCORP orthologs are restricted to insects, MCORP might be useful as a new target for insect control. Once the biochemical function of MCORP is known, it may be possible to devise a chemical method to target MCORP, and even without knowing the function of MCORP, RNAi technology could be used to target plant pests.

## Supporting Information

Figure S1 **Alignment of Insect MCORPs and MCOs for Phylogenetic Analysis.** The highly variable amino-terminal and carboxyl-terminal ends of MCORP and MCO sequences were left out of the alignment. Sequences beginning with the cysteine rich region [11] were aligned by ClustalW in MEGA5 and adjusted by eye. Gaps were omitted in the alignment for phylogenetic analysis. Abbreviations used are: Ag, *Anopheles gambiae*; Aa, *Aedes aegypti*; Cq, *Culex quinquefasciatus*; Tc, *Tribolium castaneum*; Dp, *Dendroctonus ponderosae*; Ms, *Manduca sexta*; Bm, *Bombyx mori*; Dp*, *Danaus plexippus*; Cf, *Camponotus floridanus*; Hs, *Harpegnathos saltator*; Bt, *Bombus terrestris*; Bi, *Bombus impatiens*; Mr, *Megachile rotundata*; Af, *Apis florae*; Ap, *Acyrthosiphon pisum*; Ph, *Pediculus humanus corporis*; Dm, *Drosophila melanogaster*; Am, *Apis mellifera.*
(PDF)

Figure S2 **Alignment of amino acid sequences of TcMCORP, AgMCORP, and TcLac2A.** The ten histidines and one cysteine that are expected to coordinate copper ions in TcLac2A (an MCO) are highlighted in black. Predicted signal anchors are in italicized text. A conserved cysteine-rich region is highlighted in gray. The three cupredoxin-like domains are indicated by dashed underlining (I), bold underlining (II), and wave underlining (III). NCBI accession numbers: TcMCORP, XP_967121; AgMCORP, KJ500312; TcLac2A, NP_001034487.
(EPS)

Table S1 **Primers used in this work.**
(DOCX)

Table S2 **NCBI accession numbers of sequences used for phylogenetic analysis.**
(DOCX)
