# Supplementary material for: A Multicopper Oxidase-Related Protein Is Essential for Insect Viability, Longevity and Ovary Development
Source: PLoS One. 2014 Oct 20;9(10):e111344. doi: 10.1371/journal.pone.0111344 (PMC4203857; doi:10.1371/journal.pone.0111344)
Supplement: Figure S1 — Alignment of Insect MCORPs and MCOs for Phylogenetic Analysis. The highly variable amino-terminal and carboxyl-terminal ends of MCORP and MCO sequences were left out of the alignment. Sequences beginning with the cysteine rich region [11] were aligned by ClustalW in MEGA5 and adjusted by eye. Gaps were omitted in the alignment for phylogenetic analysis. Abbreviations used are: Ag, Anopheles gambiae; Aa, Aedes aegypti; Cq, Culex quinquefasciatus; Tc, Tribolium castaneum; Dp, Dendroctonus ponderosae; Ms, Manduca sexta; Bm, Bombyx mori; Dp*, Danaus plexippus; Cf, Camponotus floridanus; Hs, Harpegnathos saltator; Bt, Bombus terrestris; Bi, Bombus impatiens; Mr, Megachile rotundata; Af, Apis florae; Ap, Acyrthosiphon pisum; Ph, Pediculus humanus corporis; Dm, Drosophila melanogaster; Am, Apis mellifera. (PDF) [file pone.0111344.s001.pdf]

```
                  ....|....| ....|....| ....|....| ....|....| ....|....| ....|....|
                       5         15         25         35         45         55
AgMCO1            CKRACTLGRK PETCYYRFRL EWYRTLSKAC YN---CPYNA TDCERPHCIT GDGVRRNVAV
AgMCO2A           CARACREGEP PRICYYHFTV EYYTVLGAAC QVC-TPNATN TVWSHCQCVL ADGVERGILT
AgMCO3            CLRECDNTQP -RICHFSWTM EHYHVMGPAC RDC-AKG-NH TDCYHPACIT ADGVERGVMS
AgMCO4            CDRICTEREA PRVCYFRWIA EHYAAMGSAC GDC-RWG-NR SHCFHPQCIT ADGMERGVLA
AgMCO5            CDRHCVPGDP PLTCHFRWKL ENYATMGSAC WDC-RLG-NR AHCFHPQCIT ANGLERGVFA
AaAAEL007802      CRRECMRGRK PMNCYYRFKM EWYETLSKAC YE---CPYNV TDCERPHCIA ADGVSRSVIV
AaAAY29698        CARACREGEP PRICYYHFTA EYYTVLGAAC QVC-TPNATN TVWSHCQCVL ADGVERGILT
AaAAEL001667      CMRSCEDTQP -RVCYFKWVL EHYHSMGPAC KRC-ADG-VH SDCYLPACLT ADGFERGVMS
AaAAEL001632      CDRVCNATET PRICYFSWVA ENYAAMGSAC KDC-RWG-NH TDCSHPQCIT ADGMERPVVS
AaAAEL001640      CDRTCSVDEP SRICYYKWVL ESYAAMGSAC WDC-IRG-NR THCFHPQCVT ANGMERSIVS
AaAAEL001672      CDRKCTVGEP SRVCYFHWVL ENYSAMGSAC WDC-LRG-NR AHCFHPQCIT ANGMERAMVA
AmLOC724890       CRRNCVDEAP PMQCHYIFRL EAYHTMSKAC YD---CPFNV TDCFRKHCIP ADGIERSILV
AmLOC410365       CARACRENEP PRICYYHFTL EYYTVLGAAC QIC-TPNATN VVWSDCQCVL ADGVERGILT
AmLOC552811       CVRNCTDNEQ PKICYYFFHI EFYTTVGPAC DIQ-GSN--- ------QCIL ADGIEKTLIP
DmMCO1            CNRDCQVGAE PMTCRYKFVV EWYQTFSKAC YD---CPRNL TDCSRPHCVM GDGLERSITV
DmLac2            CARACREGEP PRICYYHFTL EYYTVLGAAC QVC-TPNATN TVWSHCQCVL ADGVERGILT
DmMCO3            CRRVCQQG-Q SQNCYYQLVV HNYQRLGPEC QR---CQFDE RACASEHCIY GDGVANPVMA
DmCG32557         CRRDCADK-Q PMTCYYYMVV HYDDTMAETC KRYDGIALAT QLAANDDCKY ADGLESEVMV
BmMCO1            CERECKEGED PMVCYYHFNL EWYQTMSKAC FN---CPFNE TDCFRPDCIP ADGMNRPLNV
BmLac2            CARACRENEP PRICYYHFTL EFYTVLGAAC QVC-TPNATN VVWSHCQCVL ADGVERGILT
MsLac1            CERECKEGEE PMVCYYHFNL EWYQTMSKAC YN---CPFNE TDCSRPDCIP ADGMNRALSV
MsLac2            CARACRENEP PRICYYHFTL ELYTVMGAAC QVC-APNATN VVWSHCQCVL ADGVERGILT
TcLac1            CARKCVKDSV PMTCRYTFLL EWYHTLSKAC YD---CPYNT QDCYREDCIP GDGNKRSIIV
TcLac2A           CARACREGEP PRICYYHFTL ELYTVLGAAC QVC-TPNATN TVWSHCQCVL ADGVERGILT
ApLOC100165676    CQRECRAGEP PKTCEYRFKV EWYYTMSKAC YD---CPYNI TDCYRPDCVP ADGVAKPIIV
ApLOC100164049    CARACREGEP PRICYYHFTA ELYNVLGAAC QVC-TPNATN TLWSHCQCIL ADGVERGMLA
PhPHUM024710      CERPCKKG-K LMTCKYNFVI EYYSVMGKGC VD---CPFNK SHCSLKNCIP GDGIKRTVLV
PhPHUM554290      CARACREGEP SKICYYHFTL ELYNVLGAAC QVC-TPNATN TIWSNCQCVL ADGVERGILT
AgMCORP           CDRVCNRADW PRICRYELVV EKRTFRPGST PST-----DN VTLTGGSKST EQPITVSYYT
TcMCORP           CDRPCHDLDW PMICRIKIQI E----NKRPC KDC------- ---------- ---TSSEIIA
AaMCORP           CKRPCNRADW PRICRYRLVI EKISLPKFLM NHK-ISNENG AATPDGSGKL SRQVESHYFL
CqMCORP           CDRPCKRADW PRICRYRLVI EKRLMPKSLL ATT---DQAA ATTTGGHLAW QHQQHRHYFL
BmMCORP           CDRECHELDW PMICRVKLVI EVYKTLSKSC GNC------- -GECPSTCIS ADGRERGVLT
MsMCORP           CDRECHELDW PMICRVKLVI EVYKTLSKSC GNC------- -GLCPPTCIS ADGRERGVLT
CfMCORP           CDRPCHHLDW PMICRVKLTL EVFQSLSKSC GDC------- -ACLAKHCVS TDGQRRGILT
MrMCORP           CDRPCHHLDW PMICRLKLTL EVFQSLSKSC GDC------- -ACLHNHCIT ADGQRRGILT
PhMCORP           CSRPCHELDW PMICRIKLTL ERFQTLGNPC KG---CPGNI SDCYINGCIT VDGQSKGILT
BtMCORP           CDRPCHHLDW PMICRLKLTL EVFQSLSKSC GDC------- -ACLSDHCVS ADGQRRGILT
BiMCORP           CDRPCHHLDW PMICRLKLTL EVFQSLSKSC GDC------- -ACLSDHCVS ADGQRRGILT
ApMCORP           CDRECHELDW PLICRYKIVL ETQKIQNNCQ KCS-----SN NQTECQHDIY CDRFSEKIIT
HsMCORP           CDRPCHHLDW PMICRVKLTL EVFQSLSKSC GDC------- -ACLANHCVA ADGQRRGILT
DpMCORP*          CDRECHELDW PMICRVKLVI EVYKTFSKSC NSC------- --ECPAMCIT ADGRERGVLS
AfMCORP           CDRPCHHLDW PMICRVKLTL EVFQSLSKSC GDC------- -ACLADHCVS ADGQRRGILT
DpMCORP           ---------- ------MTEA S----NPR-- ---------- ---------- ------TIIT
```

	....|....|	....|....|	....|....|	....|....|	....|....|	....|....|
	65	75	85	95	105	115
AgMCO1	INRMMPGPAI	EVCENDIIVV	DVENHLMGES	TTIHWHGLHQ	RRTPYMDGVP	HVSQCPISPG
AgMCO2A	VNRMIPGPSI	QVCENDRVVI	DVENHMEGME	LTIHWHGIWQ	RGTQYYDGVP	FVTQCPIQQG
AgMCO3	LNRKIPGPTI	SVCRHDLIVV	DITNAMAGTS	AAIHWHGLHQ	RATPYMDGVP	FITQCPIGFG
AgMCO4	LNRRIPGPTI	HVCRHDLIVV	DVVNHMEGLE	STIHWHGAHQ	YDTPWMDGVP	MITQCPIPNG
AgMCO5	INRRVPGPPI	HVCKHDSIVV	DVENQLEGLG	STIHWHGFHQ	KATPWMDGVP	MVTQCPIPQD
AaAAEL007802	INRMMPGPSI	EVCENDIITV	DVENHLMGDS	TTIHWHGLHQ	KRTPYMDGVP	HISQCPISPG
AaAAY29698	INRMIPGPSI	QVCENDRVVI	DVENHMEGME	LTIHWHGIWQ	RGTQYFDGVP	FVTQCPIQQG
AaAAEL001667	INRQVPGPAI	QVCKDDLIVV	DMTNAMGGTA	TAMHWHGLHQ	RDTPHMDGVP	FVTQCPIEFM
AaAAEL001632	LNRQMPGPAV	IVCRNDIIVI	DLLNHMEGSS	TTIHWHGMHQ	TQTPWMDGVP	MVTQCPIPAG
AaAAEL001640	INRKMPGPLI	FVCQGDTIVV	DVSNEMEGMS	ATIHWHGFRQ	MQSPWMDGVP	MVTQCPIAPS
AaAAEL001672	INRKMPGPPI	FVCRGDTIVV	DVSNEMEGMS	NTIHWHGFHQ	LKSPWMDGVP	MLTQCPIAPS
AmLOC724890	VNRQMPGPAI	EVCQGDRITV	DVINLLHSES	TTMHWHGQHH	VKTPYMDGVP	YVSQCPILPG
AmLOC410365	ANRMIPGPSI	QVCQGDKVVI	DVENHIEGME	VTIHWHGVWQ	RGSQYYDGVP	FVTQCPIQEG
AmLOC552811	INRQLPGPPI	EVCLNDRVVV	DVQNAAMGME	ATIHWHGLFQ	NGFQYYDGVP	YVTQCPIASS
DmMCO1	VNRMMPGPAI	EVCEGDEIVV	DVKNHLLGES	TSIHWHGLHQ	KKTPYMDGVP	HITQCPITPH
DmLac2	ANRMIPGPSI	QVCENDKVVI	DVENHMEGME	VTIHWHGIWQ	RGSQYYDGVP	FVTQCPIQQG
DmMCO3	VNRMVPGPSI	ELCENDTVVV	DVLNYLS-EP	TTMHWHGVHM	HRTPEMDGAP	FITQYPLQPG
DmCG32557	VNGQLPGMNI	EVCYGDTVVA	DVINSMH-ET	TTIHWHGMHQ	RLTPFMDGVP	HVTQYPIEAG
BmMCO1	INRKMPGPAI	EVCQHDRVIV	DVENDLMTEG	TTVHWHGQHQ	KGTPYMDGTP	YVTQCPIPPE
BmLac2	ANRMLPGPSI	QVCENDKVVI	DVENHMEGME	VTIHWHGIWQ	RGSQYYDGVP	FVTQCPIQQG
MsLac1	VNRKMPGPAI	EVCQDDRIIV	DVENDLMTEG	TTVHWHGQHQ	RGTPYMDGTP	YVTQCPILPE
MsLac2	ANRMLPGPSI	QACENDKVVI	DVENHMEGME	VTIHWHGIWQ	RGSQYYDGVP	FVTQCPIQQG
TcLac1	VNRKMPGPSV	EVCLGDEVII	DVVNHLSSDS	TTIHWHGHHQ	KNSPYMDGVP	FVTQCPIHPG
TcLac2A	ANRMIPGPSI	QVCEGDKVVI	DVENHIEGNE	VTLHWHGVWQ	RGSQYYDGVP	FVTQCPIQQG
ApLOC100165676	INRSLPGPSI	QVCLGDTVMV	DVENAMMEES	TSVHWHGHHQ	RNSPYMDGVP	YVTQCPVPPH
ApLOC100164049	VNRMLPGPSI	QVCEGDKVVI	DVLNHMHGME	LVIHWHGIHQ	KGTQYYDGVP	YVTQCPIHEG
PhPHUM024710	VNRQMPGPRI	DVCHGDTVEV	KVTNKLMDIS	TTIHWHGILQ	KETPYMDGVP	HVSQCPIGPQ
PhPHUM554290	ANRMIPGPSI	QVCEGDKVVI	DVENRMEGQA	ASIHWHGVWQ	RGTQYSDGVP	FVTQCPIQEG
AgMCORP	VNGRYVGPTL	TVCENDFLVV	DVENRIPGES	ITLHWTGQSQ	RRTPFMDGVP	MITQCPIASF
TcMCORP	VNGQSPGPAI	QICQNDILVV	DVVNKMPGHS	LAIHWRGQPN	VEAPFMDGVP	LVTQCPILSY
AaMCORP	VNGHHTGPTL	EVCKNDFLVI	DVENRIPGRS	ISLHWTGQTQ	KRTPFMDGVP	MISQCPITSY
CqMCORP	VNGRHTGPAL	TVCERDFIVI	DIVNRIPGQS	IAIHWTGQSQ	RRTPFMDGVP	MITQCPIASY
BmMCORP	ANRALPAPTL	HVCHNDILVV	DVVHRAPAHA	LSIHWRGQPQ	KETPFMDGAP	MLTQCPQPAY
MsMCORP	ANRALPAPPL	HVCHNDILVV	DVVHRAPAHT	LSIHWRGQPQ	KETPFMDGAP	MLTQCPQPAY
CfMCORP	ANRQMPGPSI	QVCENDILVI	DVINRLPGKA	MAIHWRGQTQ	VEMPYMDGAP	LITQCPIPSY
MrMCORP	ANRQLPGPTI	QVCENDILVI	DVINRLPGKA	AAMHWRGQSQ	VESPFMDGAP	LITQCPIPSY
PhMCORP	VNRQFPGPTL	EVCQYDIVLV	DIVNRIPGQS	FGVHWRGQSQ	SETPFMDGVP	MITQCPIPSL
BtMCORP	ANRQLPGPII	QVCENDILVV	DVINRIPGKT	AAMHWRGQTQ	IETPHMDGAP	LVTQCPIPSY
BiMCORP	ANRQLPGPII	QVCENDILVV	DVINRIPGKT	AAMHWRGQTQ	IETPHMDGAP	LVTQCPIPSY
ApMCORP	ANRQVPGPSI	RVCENDIMVI	DIVNRIPGHS	VSVHWRGQWQ	KETPVMDGAP	MVTQCPILPH
HsMCORP	ANRQMPGPSI	QVCENDILVV	DVINRLPGKA	TAVHWRGQTQ	LEMPYMDGAP	LVTQCPIPSY
DpMCORP*	ANRELPAPAF	HVCQNDILVV	DVVHRAPAHA	LSIHWRGQPQ	KETPFMDGAP	MLTQCPQPAY
AfMCORP	ANRQMPGPTI	QVCENDILVV	DVINRLPGKA	AAIHWRGQSQ	LETPYMDGSP	LVTQCPIPSY
DpMCORP	ANRQIPGPPI	QVCQNDILIV	DVINRVPGKS	VTLHWRGQPN	HEAPFMDGVP	MVTQCPIPSY

	....|....|	....|....|	....|....|	....|....|	....|....|	....|....|
	125	135	145	155	165	175
AgMCO1	TTFRYTFRAD	NPGTHFWHSH	TGMQRGDGAF	GALIIRK--D	NDIQELLYDH	DLSEHVITVQ
AgMCO2A	NTFRYQWTG-	NAGTHFWHAH	TGLQKLDGLY	GSIVVRQPPS	RDPNSHLYDF	DLTTHIMLVS
AgMCO3	NTFRYAFLAT	EPGTQFYHSH	SGHHKVNGHY	GALIVREPKR	VDPNGDLYHY	DTPAHVILGS
AgMCO4	AAFRYAFNAS	EPGTQLYHSH	SGHQKANGHY	GLFVIRSP--	TDINRHLYDY	DLSEHHIITS
AgMCO5	TTFRYQFTAV	EAGTQFYHSH	AGFQKANGHY	GMVVVRDP--	SDLNQAHYDY	DLSEHRIIIA
AaAAEL007802	TTFRYTFKAD	NAGTHFWHSH	TGMQRGDGAF	GPLIIRR--D	NDPQQILYDH	DLSEHVITVQ
AaAAY29698	NTFRYQWTG-	NAGTHFWHAH	TGLQKLDGLY	GSIVVRQPPS	RDPNSHLYDF	DLTTHIMLVS
AaAAEL001667	STFRYAFWAT	EPGTQFYHSH	AGHHKVNGHY	GAMIIRQPEV	NDPNAKLYDF	DLPEHLIVAS
AaAAEL001632	NTFRYVFNAS	EHGTQFYHSH	AGHQKANGHF	GLLVVRHP--	TDLNMNLYDY	DLSEHHIIIA
AaAAEL001640	TTFRYRFVAE	EAGTHWYHSH	SGYHMANGHL	GAAVVRNP--	LDVNMALYDF	DLSEHVMLIS
AaAAEL001672	SSFRYTFQAE	EPGTQWYHSH	AGYHMANGHL	GVAVVRNP--	LDVNADLYDF	DLSEHVILLS
AmLOC724890	STFRYDFIAT	EAGTHFWHSH	SGFQRGDGVF	GPLIVRTPPK	ANWHKDLYDI	DE--HIIQIS
AmLOC410365	STFRYQWTAG	NEGTHFWHAH	TGLQKMDGLY	GSIVIRQPPS	KDPNSNLYDY	DLTTHVVLIS
AmLOC552811	STFRYDFVVK	NSGTHFYHSH	ISTHMLDGQI	GSFIVKDPPR	KNPHRDLYDK	DE--IVIFLS
DmMCO1	ATFRYSFPAD	LSGTHFWHSH	TGMQRGDGVF	GALIIRKPKT	AEPHGGLYDF	DLSEHVMIVQ
DmLac2	NTFRYQWTG-	NAGTHFWHAH	TGLQKLDGLY	GSVVVRQPPS	RDPNSHLYDF	DLTTHIMLIS
DmMCO3	EVQRHEFKVD	RSGSLWYHSH	VGWQRGFGVA	GAFVVRQTSQ	ENQHSQLYDY	DLVEHTLMIQ
DmCG32557	QAFRYRFEVD	HGGTNWWHSH	TEHQRAFGLA	GPLVVRMPPK	LNPHAHLYDF	DMSEHVIMIQ
BmMCO1	TTFRYQFNAT	HTGTHFWHSH	SGMQRADGAA	GAFIVRKPKS	QDPHGHLYDY	DRTDHVMIVT
BmLac2	NTFRYQWQG-	NAGTHFWHAH	TGLQKLDGLY	GSIVVRQPPS	KDPNSHLYDY	DLTTHVMLIS
MsLac1	TTFRYQFTAR	HSGTHFWHSH	SGMQRADGAA	GAFIIRKPKS	QEPYESLYDY	DRSDHVMIVT
MsLac2	NTFRYQWQG-	NAGTHFWHAH	TGLQKLDGLY	GSIVVRQPPS	KDPNSHLYDY	DLTTHVMLIS
TcLac1	MTFRYHFNVH	NSGTHFWHSH	SGFQRSDGTF	GPFIVRVPEE	DNPHAKLYDY	DLSSHVITIL
TcLac2A	NTFRYQWIAG	NAGTHFWHAH	TGLQKMDGLY	GSVVIRQPPA	KDPNSHLYDY	DLTTHVMLLS
ApLOC100165676	SSFRYVYLAD	NEGTHFWHSH	SGCQRGDGAF	GSFVVRAPKS	RDVHRDMYDV	DV--HVITVT
ApLOC100164049	NTFRYQFDT-	NSGTHFWHAH	SGLQKIDGIY	GSIVVRQPPS	QDPNSHLYDY	DLTTHVVLLS
PhPHUM024710	SSFLYKFYAD	SPGTHIWHAH	SAFQRGDGIY	GGLVVRVPPE	ENRHLSLYDF	ELSEHVFTVM
PhPHUM554290	NTFRYQWNAE	NAGTHFWHAH	TGLHKLDGLY	GSIVIRQAPS	KDPNSHLYDY	DLTTHVMLLS
AgMCORP	TRFQYKFQAD	RAGTHLYHGF	AGSERTQGLL	GAFVVRSAYE	QRQSPVLSAL	-HNDPVWLVT
TcMCORP	TTFQYKFRVT	SPGTHLYQAF	SDSELDRGLF	GALIVRQAEK	NDLQRKYYDV	DSRNHIIMIS
AaMCORP	TTFQYKFQAN	RVGTHLYYGF	SNDERKLGLI	GALLVRSVHE	QSQHPLTSQC	-QDDLIWLIS
CqMCORP	TTFQYKFQAD	HVGTHLYHGF	SAEERGLGLV	GAFVVRSEHE	QRIHPVTSGC	HQQELVWIIA
BmMCORP	TTFQYKFRAS	AVGTHMYHAH	SAADAADGLA	GALVVRQSPR	QDPLRKLYDT	DASEHTIYVS
MsMCORP	TTFQYKFRAS	AVGTHMYHAH	SAADAADGLA	GALIVRQSKR	MDPLSKLYDI	DSTEHTIFVS
CfMCORP	TTFQYKFRAS	MPGTHLWHAH	AGADITNGIF	GALIVKQADL	REPHRALYDI	DDPNHVVLVT
MrMCORP	TTFQYKFRAS	VAGTHLWHAH	AGADVTNGIF	GALIVKQADI	KDPHRSLYDI	DDSNHVVLVS
PhMCORP	TTFQYKFRAS	EPGTHIWQVN	TGEEYLDTLF	GPLIVKKPYS	KEINKNYYDT	DDKKNVVVIH
BtMCORP	TTFQYKFRAS	SAGTHLWHAH	AGADVTNGIF	GALIVKQADI	KDPHRALYDI	DDSDHVVLVS
BiMCORP	TTFQYKFRAS	SAGTHLWHAH	AGADVTNGIF	GALIVKQADI	KDPHRALYDI	DDSNHVVLVS
ApMCORP	TTFQYKFRAA	QAGTHWWQIL	SGDELSDRVY	GSFIVKQSKR	REPHASIYDY	DEIPHVLLVE
HsMCORP	TTFQYKFRAS	VPGTHLWHAH	AGADVSNGIF	GALVVKQADL	REPHRALYDI	DDLNHVVLVT
DpMCORP*	TTFQYKFRAS	AVGTHMYHAH	SAADAADGLA	GAFIVRQSPR	LDPLASLYDV	DATDHTIFVA
AfMCORP	TTFQYKFRAS	AAGTHLWHAH	AGDDVSNGIF	GALIVKQADI	RDPHRALYDI	DDPSHVILVS
DpMCORP	TTFQYKFRAS	KPGTHFYHAY	MDADRSNGLF	GALIVRKSDR	TEPSKKMYDV	DSKDHYILIS

```
                  ....|....| ....|....| ....|....| ....|....| ....|....| ....|....|
                        185        195        205        215        225        235
AgMCO1            DLLINGRGKH VDKGRRYRFR LINAEFLNCP VELSIENHNL TVIASDGFGI QPLGSFVSYA
AgMCO2A           DLLINGKGQT ITPGRRYRFR MINAFASVCP AQVTIEGHAL TVIATDGEPV HPVNTIISFS
AgMCO3            DLLINGKGTT VRRGARFRFR FINAASHVCP LQLQIEDHMM EVIASDSFHL QPVDTLVSTS
AgMCO4            DILINGRGRR VKKGYRYRFR LVSSGSQFCP FQLQIEKHRM QLIVTDGGAV QPVDTLISTS
AgMCO5            DILINGRGRQ VEYGKRYRFR LISSGSQYCP FQMQIQNHSM LIISTDGGTV QPVDTLVSIS
AaAAEL007802      DILINGRGKH VEPKKRYRFR LINAEFLNCP VELSVEGHNL TVISSDSFDI NPLASIVSYA
AaAAY29698        DLLINGKGQT ITPGRRYRFR MINAFASVCP AQVTIEGHGL TVIATDGEPV LPVNTIISFS
AaAAEL001667      DLLINGRGTR VRKGGRYRFR FINAASHVCP LELQIANHTL EIIASDSYNL QPANTLVTTS
AaAAEL001632      DILINGRGRR VEQWKRYRFR MISSGSQFCP FQLQIEAHRM QIISTDGGAV QPVDTVISTS
AaAAEL001640      DILINGRGRR VRRNYRYRFR LISGGSQYCP FQLQIENHRM LVISTDGGAV KPVDTLISIS
AaAAEL001672      DILVNGRGR- ---------- ---------- ---NIEKHQK TIISTDGGAV KPVDTLISIS
AmLOC724890       DILINGLGRT VKQNTRYRFR LVNAEFLNCP IEISIDNHTM RVISSDGRDI EAAESLVSYA
AmLOC410365       DVLINGKGQT ITPGRRYRFR LINSFGSVCP SQITFEGHSL TIIATDGEAV QPVDTIISFS
AmLOC552811       DILINGLGNT VKKGERHRIR MINSFSTVCL TELRIEKHKL IIIAQDGENV KPVDKIVTST
DmMCO1            DLLVNGKGRT VRRGFRYRFR IINAEYLNCP IVVSIDGHNL TAINSDGFDI EAVGSIVTYS
DmLac2            DMLINGKGQT ITPGRRYRFR MINAFASVCP AQVTIEGHGM TVIATDGEPV HPVNTIISFS
DmMCO3            DILVNGKGRR VTPGYRYRMR VILNGIANCP VEFSIEQHRL LMISTDGNDI EPADGFFLTS
DmCG32557         DILINGRGRP VVRGGRYRFR VIFNGVSNCP ISFSIDKHDL VVIASDGNDI EPVQRIMFHG
BmMCO1            DLLINGVGRN VEQGHRYRFR VINAEFLNCP IELSVDGHNI TVISSDGYDL EPATSLVTYA
BmLac2            DVLINGKGQT ITPGRRYRFR MINAFASVCP AQITFEGHNL TVIATDGEPV QPVNTIISFS
MsLac1            DLLINGVGRN VEQGYRYRFR VINAEFLNCP IEMSVDGHNI TVIASDGYDL EPATSLVTYA
MsLac2            DVLINGKGQT ITAGRRYRFR MINAFASVCP AQVTFEGHNL TVIATDGEPV QPVNTIISFS
TcLac1            DILVNGFGRT VEQGYRYRFR VINAGFLNCP IEVSIDNHTL SVISTDGSDF NAVDSLVTYA
TcLac2A           DLLINGKGQT ITPGRRYRFR MINSFASVCP AQLTIQGHDL TLIATDGEPV HPVNTIISFS
ApLOC100165676    DILINGRGRN VTRGKRYRFR LINAGFLNCP ISMSIDNHTF TVIATDGYNV QPVDSFVSYA
ApLOC100164049    DLLINGKGQT ITPGRRYRFR MINALASVCP AQITIQGHPL VLIATDGEPI QPVNTIISFS
PhPHUM024710      DLLINGKGRT VKQGYRYRFR LMNTGFLNCP IEMSIDEHNI TVISSDGEDL QPTVSLVSLA
PhPHUM554290      DLLINGKGQT ITPGRRYRFR MINSMASVCP VQLTIQGHSL ILIATDGEPV HPVNTIISFS
AgMCORP           ELGVNGQRD- LNGTLNLRVR LTYAV---CQ HWLELEDHRL QVLALDGNVL DHVSRILLHD
TcMCORP           EVLVNGKG-T VKRNKRYRFR VAFAGNSGCP VTLTVDNHLI KVIALDGNLV FPVTSVVLTK
AaMCORP           EFLINGNRS- VQPKSRYRLR VAYAANH-CQ RWLEIQDHNL TVIALDGNLV EPVERVALSD
CqMCORP           ELSINGRRS- AKPGLRYRLR VAFVSPEGCQ HWLEVDQHRL TVIALDGNLL EPVERVALSD
BmMCORP           ELLINGKGKS VEYGKRYRFR LAYGGSKSCP IQFSIEKHVL TLVALDGNGI EPVNSIELGR
MsMCORP           ELLINGKGRT VEHGKRYRFR LGYGGSKSCP IRFSIEKHVL ELVALDGNRI STVNSIGLGR
CfMCORP           HLLINGRGRT VIPGRRHRFR VANAGAGACP VTLFIDSHTL LLIALDGHPI EPVTSITLAK
MrMCORP           QLLINGRGRT VVPGRRHRFR IANAGAGSCP ITISVDAHPM LLIALDGQPV EPVTSITLAK
PhMCORP           TLLINGKTQN VTAGKRHRFR VIYVGEKNCQ IRFSIDEHKF FVIGFDGKSI QPVTSVKLFP
BtMCORP           QLLVNGRGRT VIPGRRHRFR VANAGAGSCP ITISVDAHPL LLIALDGQPV EPIASITLAK
BiMCORP           QLLVNGRGRT VIPGRRHRFR VANAGAGSCP ITISVDAHPL LLIALDGQPV EPIASITLAK
ApMCORP           YMRINGVEST VQSNSKYRFR TINTGVSQCP IEIKVHKHHL TVIAIDGHAI EPVDVIQVEP
HsMCORP           QLLINGKGRT VVPGRRHRFR VANAGAGACP VTLFIDGHSL LLIALDGHPI EPVTSITLAK
DpMCORP*          ELLINGKGKN VEYGKRYRFR LAYGGFKSCP INFSIDKHAI KLVALDGHII QTVTSIELGR
AfMCORP           QLLVNGRGRT VLPGRRYRFR LANAGAGSCP ITVLLDAHPL LLIGLDGQPV EPVASITLAK
DpMCORP           ELLVNGKA-N VKPGRRHRFR VAYTSLSGCP VNLTVDNHLL KIIELDGNPT NPVSSIRISK
```

```
                    ....|....| ....|....| ....|....| ....|....| ....|....| ....|....|
                         245        255        265        275        285        295
AgMCO1              GERFDFIVKA NQPIGNYLIR FRGLMDCDER FTSAYQFAVL RYRGAPT--A PGVQLNSLNR
AgMCO2A             GERYDFVITA DQPVGAYWIQ LRGLGECG-- IKRAQQLAIL RYARGP---P QGVVMNPLD-
AgMCO3              GERYDFVLEA NGVKDTYWVR LRSLGPCA-- DLQLEQFAVL RYTTG--PFR NVATANHPN-
AgMCO4              GERYDFVLSA NQKPGTYWVR VRAIGFCN-- IERREEFAVL SYEDEAHHVP SGTVLNNPN-
AgMCO5              GERYDFVLTA NQPPGNYWVR VRGIGFCD-- QMRVEDFAIL SYRTPETAIP DGIVFNHQT-
AaAAEL007802        GERFDFILRA NQPVGNYLMR FRGLMDCDER FTSAYQVAVL RYKGAP---L EGMQLNSLNK
AaAAY29698          GERYDFVISA DQQVGAYWIQ LRGLGECG-- IKRAQQLAIL RYARGP---P QGVVLNPLD-
AaAAEL001667        GERYDFVVNA DQPTDDYWIR LRAIGPCD-- YRQISQVAVL SYQPMSVPEL NDVYVNHPN-
AaAAEL001632        GERYDFVLHA DQKPGDYWVR VRAVGFCN-- IQRKEEFAVL SYRSSSE-IP DGMTLNHPN-
AaAAEL001640        GERYDFVINA DQPVGNYWVR VRGAGFCS-- TLSVETFAIL SYADPSISTP MGQTLNEQT-
AaAAEL001672        GERYDFVLTA NQPPGNYWVR VRGIGFCN-- SQRVEGLAIL SYADSSIPTP LGRTLNHHM-
AmLOC724890         GERFDFVVET SQNIDNFWIR FRGLMDCDER FTKAYQVAIL RYEGATN--T DGQRINALNE
AmLOC410365         GERYDFVINA DQPVGAYWIQ VRSLGECG-- IPRAQQLGIL RYARGP---P QGVVLNPLD-
AmLOC552811         GERVDFILVA NQSVDSYWIQ ARGLGECA-- TTFMQQLAIL KYENGP---- DGVIYNGLNG
DmMCO1              GERFDFVLNA NLEVGNYWIR LKGLMDCSEV FTSAFQVAIL RYEGAPD--- EGIELNVMNR
DmLac2              GERYDFVISA DQPVGAYWIQ LRGLGECG-- IRRAQQLAIL RYARGP---P QGVVMNPLD-
DmMCO3              AERFDFVLEA NQYKKNYWIR IKGYEQCE-- NRNIYQGAVL SYRGSARS-E DLILVNDFRF
DmCG32557           AERFDFVLHA NQEVSNYWIR VKGYSFCA-- KNQLHQEAVL HYRDADTR-A PGKTLNELGD
BmMCO1              GERYDFILDA NNEIDNYWIR FRGLMDCDEV FTKAKQVGVL HYEGAME--N EGLQLNALNK
BmLac2              GERYDFVIEA NNIPGAYWIQ VRGLGECG-- IKRAQQLGIL RYARGP---P QGVVMNPLD-
MsLac1              GERYDFILDA NNEIDNYWIR FRGLMDCDEI YTRAKQVAVL HYEGAMD--N EGLQLNALNK
MsLac2              GERYDFVIEA NNIPGAYWIQ VRGLGECG-- IKRAQQLGIL RYARGP---P QGVVMNPLD-
TcLac1              GERFDFIVTA DQPQDVYWMH FRGLMDCDER FTRAYQVAVL EYKGTQT--R EGKQLNPLNK
TcLac2A             GERYDFVINA DQTPGAYWIQ LRGLGECG-- IRRVQQLGIL RYAKGP---P QGVVLNPLD-
ApLOC100165676      GERWDFVVEA TANVGNYWMR FRGLMDCDER FTKAFEVSIL HYDGAGD--H SGIQLNALNK
ApLOC100164049      GERYDFIINA EQPVGAYWIQ VRGLGECG-- NKRVQQLAIL RYARGP---P QGVVMNPLD-
PhPHUM024710        GERFDFVVEA NKKIDNYWIR FKGLLDC--K FKEAYQTAIL HYDGADID-R TGLQVNKVNS
PhPHUM554290        ---------- -----AYWIQ ARGLGECG-- IARAQQLAVL RYARGP---P QGVVLNPLD-
AgMCORP             GVRMDVALTR L---RDYEIR FTPHGTTSSC GRAVFRLHYD AERAAANDAH RSEELQLDLT
TcMCORP             GERLDFVLKT DQKIKRHYVR VKS--CLG-- ----EGLALL NYELG----- -------RVL
AaMCORP             GNRVDLIVHT N---QDYEIR LVATGDSPRS -------VYT GYNSNECHLG STNNFRLKYV
CqMCORP             GDRIDLILET SGAGQEYEIR LVSVAS---- --------YN G-SCEQEHL- SSSRFKLKYV
BmMCORP             GERVDFILEA KRAPGVYKMS VVAHPDCQD- -N-LKGVAEL VYTNK----L NKVYRKFSTV
MsMCORP             GERADFVLEA KMIPGVYKIS VIADESCQN- -N-LEGIAEL VYYKN----N EHVTREFTTV
CfMCORP             GLRADFVLKA SKHVASYWMN VHTTKECGT- -STINGAAIL NYKGS-SEII GMNQLAAMTN
MrMCORP             GERADIVLKA NKRVASYWMH VHTSKECGT- -SPINGAAIL SYKGSTMEST S--RVTMTTN
PhMCORP             GERFDFVLSA NQKMASYSMK ATPVQCLN-- --LLSTSALI QYEGNEKKNP EFFEISTSFD
BtMCORP             GERADFVLKA NKRVASYWMN VHTSKECAV- -SPINGAAIL EYKGSSTKDV G---VAMTTN
BiMCORP             GERGDFVLKA NKRVASYWMN VYTSKECAI- -SPINGAAIL EYKGSSTKDV G---VAMTTN
ApMCORP             GETLDFILTT IKNQGIYDMT VTSEGHCK-- DSNHTHTLYI HYNSTLYN-- ---TISDTDN
HsMCORP             G-RADFVLRA SQRIGSYWMH VHTAKECGT- -STVNGAAVL NYKGS-TEVV ETTRLAATTN
DpMCORP*            GERADFILDA NQAIGVYKIR VVADKSCQD- -D-LEGEAEL IYKNQ----D STINRIFSTV
AfMCORP             GNRADFVVKA NKRIASYWIN VHASKECGT- -SPINGAAIL SYKGSSTEDS ND-RVAMTTN
DpMCORP             GERIDFILKA SQEIGAYYLS VKS--SCES- -SDLHGLAVI NYEGR----G EKNTKLKRHF
```

	....|....|	....|....|	....|....|	....|....|	....|....|	....|....|
	305	315	325	335	345	355
AgMCO1	GP--GAENVI	TIAETSALDQ	ED--LLLLRN	ETDYKFYVYY	DFYGKDNPHQ	QVVNNTNRLY
AgMCO2A	AQCNVQRDD-	AICVSQLKNA	KE-DRALLQD	KPDVKIFLPF	RFYLYRPEEF	LVAPTG-DHV
AgMCO3	ATCGRPEFG-	DYCITDFQAY	DT-DEDVING	VPDHQLTFGF	YNYPVSFESY	MNIYGS-VMM
AgMCO4	ATCYVPGDD-	DLCVADLESH	EVHDDDLIDA	APNKTFRILF	NTFTADPAVY	MTVVLT-LNN
AgMCO5	APCYTPNDT-	YICAADLEAY	EVFDTGLIDA	VPDRTFFLGF	HVIEANNSLY	ATVREG-FNT
AaAAEL007802	GT--GHSDTM	SIAETSSRDQ	ED--LLLLRE	KTDFKFYVYY	DFYAKDNPHK	DVINNTNRLF
AaAAY29698	AQCNVQRDD-	AICVSQLKNA	KD-DRALLQE	KPDVKIFLPF	RFYLYRPEEF	LVAPTG-DHV
AaAAEL001667	TTCGVSKP--	DVCITDFQAY	ET-DDDVING	VPDMQFILGF	ENYPMKFEDF	MNIHDD-IVL
AaAAEL001632	STCYQPGDQ-	FVCASDLEAH	EVQDDALIDA	VPDKKFYVAF	NTFTADTSLY	MTVALT-LNN
AaAAEL001640	ATCYNSEDQ-	FTCAADLETH	EAHDDKLINA	TPDVRLFLGF	KMMHPDNRWF	ITVRED-FNT
AaAAEL001672	TECYKEGDD-	FTCAADLEAH	EVHDHELIDS	HPDVRLFLGF	KVIEANNSMY	ATVRNK-FNT
AmLOC724890	GT--ETNNSI	SIPLLKAMDK	ND---KSNTA	DPDYQFYVSY	DFYKKDNPHN	QVKN-TKQVL
AmLOC410365	AICNRQRED-	AICVSQLKNA	RQ-DQGILQQ	RPDVKIFLPF	RFLFYRPEEF	LVAPTG-DHV
AmLOC552811	TLCNTNITEP	VLCINQLESL	ES-ND-LLKV	EPDERHILPF	WFFNYTDTSF	FNANDR-SQL
DmMCO1	GP--GYPDTK	TVAEMRALPI	YDHDHDTLKP	EADYKFFIYY	DFYTKNNPDD	MEMTQQNRLY
DmLac2	AQCNRQRND-	AICVSQLKNA	LE-DRGILAE	KPDVKIFLPF	RFFVYRAEDF	LVAPTG-DHV
DmMCO3	KP----ANST	AISSLRQSLD	KD-------N	NVGTVALRSV	DPVPWTRYTG	SRTAPNGE-V
DmCG32557	DA----SGAR	AG--------	---------N	SISLANLNAQ	RPEPEVAPSN	AFEVRQGEGF
BmMCO1	GE--EEDETI	SVAEMKSLDG	YD---ESLKE	IADYQFYVAY	DFYAKNNSHY	QVPEKVNRLY
BmLac2	ARCNISRND-	AICVSQLKNA	QN-DPAILQE	RPDVKIFLPF	RFFVYRPEMY	LVAPGG-DHV
MsLac1	GE--EENETI	SVAEMRSLAG	YD---DSLKE	IADYQFYIAY	DFYAKNNSHY	QVPEQVNRLY
MsLac2	ARCNILRND-	AICVSQLKNA	KH-DPAILQE	RPDIKIFLPF	RFFVYGPETY	LVAPSG-DHV
TcLac1	GT-EADSSFV	TLPQLHSLDE	WD---DTLKE	KADFQYYVSY	DFYKMNHPVH	NVTNTTLQNL
TcLac2A	ARCNEIRPD-	AICVSQLKNA	LS-DKGILRE	KPDVKIFLPF	RFHIYTPEDH	LVAPNG-DHV
ApLOC100165676	GSGLMDTATV	SELEDATPPK	ND---LRLEK	KPDVTLFMSY	DFYSLDNPHK	QVTHRSEQVY
ApLOC100164049	AVCDRPRTD-	AICVNQLKNA	KV-DKGLLQE	RPDVKIFLPF	KFLFYRPDEY	LVAPGGGDHV
PhPHUM024710	RG--GHENYL	IISELSSLPD	KYQ--PALKP	EPDFRFYIPF	DFYPLDSDYP	----GTQRPG
PhPHUM554290	AVCNRERSD-	AVCVSQLKNA	KP-DEGILQQ	RPDVKIFLPF	RFLFYRPEEY	LVAPSG-DHV
AgMCORP	GTACGDTMLC	P------QDL	PG-LPKDLQH	GYDTRLEFTI	GTRQQAGTG-	--FGEPTHET
TcMCORP	DTSLCHSQIG	KVCLSDLNFV	EKM-ETVK--	TAPKTVYLSL	GSQIVN----	--VSGNFGSR
AaMCORP	RSETADSTLP	P------SGA	TGDFPPELRE	-VERQISLVL	-TKRKVERQ-	--FGERFYDV
CqMCORP	GTEAAGTQIL	MKEPDDAGDA	RGTFPAELRD	-VDKTIRLVL	-TKRAVQRE-	--LGELFQDI
BmMCORP	ISVQCEDE-S	VLCLTEARGY	EQM-SELTKT	-PDRTLYVPF	NYSTRRISA-	-RVESWGQS-
MsMCORP	FSDRCTAE-N	VLCLTDVHSL	DKL-ADLVSY	-PDKIIYVPF	NYSTRQISA-	-RVESWGQT-
CfMCORP	PIEKCGGQ-E	SLCVMDMQNI	RKM-RALAMP	KMDVTLYLPI	NYKM-QATE-	-IGNRGTETR
MrMCORP	PAEKCENP-E	SLCVTEIQAL	RKI-SVLAKP	RTDVTIRLPI	NYKF-QAND-	-VVN-GAETR
PhMCORP	EDCKSDEFS-	RICIQNIRSY	EKI-KELAIE	KPDFKYYLII	DSTMSTNHN-	--IGMKETVR
BtMCORP	PAAKCENP-E	SLCVTELHAL	RKI-AALGQP	KTDVTIRLPI	NYKL-QTSD-	-VGNSGVEMR
BiMCORP	PAAKCENP-E	SLCVTELHAL	RKI-AALGKP	KTDVTIRLPI	NYKL-QTND-	-VGNSGVEMR
ApMCORP	LAKPAEYGER	HLSITSLDSL	PY---ELSAV	ELKNTIYLGF	SSIKYQLGE-	-------SWS
HsMCORP	PVDKCGGR-E	NLCVMDVQNV	RKM-HVLAKP	RMDVTMYLPI	NYRVNQETE-	-IDN-GGATR
DpMCORP*	ASDNCVSD-T	VLCLDEIHGA	EKL-SELAEP	-VDEVLYVPF	NYSTRQMSA-	-RFESWGQT-
AfMCORP	PADKCGNP-G	SLCVTELHSL	RKM-SGLGKQ	KTDVTVRLPI	NYKL-QTSD-	-VGERFVGTR
DpMCORP	DTSLCRTESG	KVCLGDVKSL	DKM-KELRKE	TVDRNIFLAI	DYKYGERET-	-EQYADLRKK

	....|....|	....|....|	....|....|	....|....|	....|....|	....|....|
	365	375	385	395	405	415
AgMCO1	TPQLNHISMR	MPPVPFLP-C	SHVLQIPLHA	TVEMVMIDNH	PFHLHGHAFR	VVGMDRVSRN
AgMCO2A	ISLIDEISYL	SAPAPLLSQC	THKVDIPLNA	IVEVVLVDSH	PFHLHGYAYN	VVGIGR-SPD
AgMCO3	QGAINNISLA	YPPFSLLTQC	THRVKINLGD	IVELYILDNH	PFHLHGYQMF	VMEMSQDRRV
AgMCO4	IGVTNNISMV	FPDFPLLTQC	LHRLKVALND	VVEMSLIDYH	PFHLHGHRFI	VTGMGQLP-Q
AgMCO5	IGATNMISFV	PPSFPLLIQC	THRMKVKHND	VIEIVLYDYH	PFHLHGHRFI	VTDSGSFPSD
AaAAEL007802	TPQLNHISMR	MPKIPMMP-C	SHVVQVPLNS	TVEMVLIDNH	PFHLHGHAFR	VVGMERLAGN
AaAAY29698	ISLIDEISYL	SAPAPLLSQC	THKVDIPLNA	IVEVVLVDSH	PFHLHGYAYN	VIGIGR-SPD
AaAAEL001667	QGAINNISFT	YPPFSLLTQC	IHRLKIPLHA	LVELYILDNH	PFHLHGYQMY	VMEMGQDRST
AaAAEL001632	IGITNNISMV	YPSFPPLTQC	VHRLKVDLND	IVEMSLIDYH	PFHLHGHRFI	VTGMGQLPTR
AaAAEL001640	IAAANNISFR	YPSFPLLIQC	THRIKVKLND	IVELTLYDYH	PFHIHGHRFI	ITDMGRLPES
AaAAEL001672	IGVANNISFI	SPSFPLLIQC	THRLKVKHNV	LVEFVLYDYH	PFHLHGHRFI	ITDMGMLPDE
AmLOC724890	TPQLNHISMK	LPPMPLLS-C	THVLRVNLDS	VVEIILVDNH	PFHLHGYQFR	VIAMERIGEN
AmLOC410365	ISLVDEISFT	FPPAPPLSQC	THQVDIPHNA	VVEVVLVDSH	PFHLHGYAFN	VIGIGR-SPD
AmLOC552811	LSIFNDIAYE	NPASNLLTQC	AQIIKTKLNN	VVELVMYDDH	PFHLHGFAFQ	VFSVGQFWPI
DmMCO1	TPQLNHITLN	FPSLALLP-C	HHVLQVPLGA	VVEMIIVDNH	PFHLHGNAFR	VMGLERLGEN
DmLac2	ISLIDEISYL	SAPAPLTSQC	THKIDIPLNA	IVEVVLVDSH	PFHLHGYGFS	VIGIGR-SPD
DmMCO3	LFQISDISYN	SPGISLLQGC	VNVMRLPAYR	PLEMVVANTH	PFHIHGFTFR	LVGQGVLGNL
DmCG32557	RFQMDDISFS	MPKMSLLQTC	SNVIQVPANQ	QVEFVISSPH	PIHLHGYTFR	VVGMGVLGEQ
BmMCO1	TPQLNHISMK	MPSSPLLV-C	SHVLSVKLNS	VVEVVIVDNH	PFHLHGHSFR	VVGLRRLASD
BmLac2	ISLIDEISYM	SPPAPLISQC	THKVDIPLNA	VVEIVLVDSH	PFHLHGYSYN	VIGIGR-SPD
MsLac1	TPQLNHISMK	MPTSPLLI-C	PHVLSVKLNA	IVEVIIVDNH	PFHLHGHSFR	VVGLRRLNRT
MsLac2	ISLIDEISYM	SPPAPLLSQC	THKVDIPLNA	VVEIVLVDSH	PFHLHGYAYN	VIGIGR-SPD
TcLac1	TPQLNYISMK	LQSFPLLS-C	THVVNIPLGT	VVEMVLIDNH	PFHLHGHSFR	VVAMERVGSH
TcLac2A	ISLIDEISYM	APPAPLISQC	THKVDIPLNA	IVEIVLVDSH	PFHLHGYAFN	VIGIGR-SPD
ApLOC100165676	TPQINKMSFK	LPSFPLLS-C	TNIIKVPLGS	IVELFLIDNH	PFHLHGHPFR	VVAMERVGNH
ApLOC100164049	ISLVDEISYT	SPGSPMISQC	SHKVDIPRHA	VVEVVLVDSH	PFHLHGYSFN	VIGMGR-SPD
PhPHUM024710	TPQLNHISME	FPSFPIMT-C	IHVLQVPIDS	VVEIVLIDNH	PFHLHGIKFR	VLGMDRLGNN
PhPHUM554290	ISLVDEISYV	APPAPPLSQC	THKVDIPLNA	VVEVVLVDSH	PFHLHGYAFN	VIGIGR-SPD
AgMCORP	VRSVNGLTFA	FPPVLMVRDC	VHVEHIEAGH	RVEMVLINDY	VYHLHGQSVF	VVA---LAGR
TcMCORP	VFGVNNLTFT	YPSSPLLTQC	VHVEHIPLRA	VAEIVLINEH	IFHLHGYRFY	VVGFRHFENA
AaMCORP	SYSVNGFSFI	YPSVLMLQRC	VHVESVELGH	RVELVIVNAY	TYHLHGYSFF	VIASTRTTQH
CqMCORP	SYSVNGFSFV	FPTAVMLRKC	VHVEHVEAGH	RVELVLINDH	SYHLHGHSFY	LVG--AAHFE
BmMCORP	----DGHRFT	YPASPLLTQC	VHVKNIPLYS	TVEIVMFDDH	IFHLHGYGFY	VTGVREFNRS
MsMCORP	----DGHRFT	YPASPLLTQC	VHVKNIPLHS	TVEIVMFDDH	IFHLHGNSFY	VTGIREFNTS
CfMCORP	VLNVNNVTFT	YPSSPLLTQC	VHVRHVPLGA	TVEIILLDDL	VYHLHGYSFY	IVGARQFGRS
MrMCORP	ILNVNNVTFT	YPSSPLLTQC	VHVRYIPLDS	TVEIILLDDL	VYHLHGYNFY	IVGARKFDRS
PhMCORP	IKQINNLTML	LPSSPLVSQC	VHVVKIPLNS	VVELVLVNDH	VFHLHGYSFR	IVGISKVPTF
BtMCORP	VLNVNNATFT	YPSSPLLTQC	VHVRYIPLGA	TVEIILLDDL	VYHLHGYTFY	VVGARKFGRS
BiMCORP	VLNVNNATFT	YPSSPLLTQC	VHVRYIPLGA	TVEIILLDDL	VYHLHGYTFY	VVGARKFGRS
ApMCORP	LPNFNNMTMV	LPSAPLLLQC	THIIDLPLGS	ATELVIFDSH	SFYLHGHSFY	VVGQKSKAFV
HsMCORP	VLNVDNATFT	FPSSPLLTQC	VHVRHVPLGA	TVEIILLDDL	VYHLHGYSFY	VVGARQFGRS
DpMCORP*	----DGHRFT	YPASPLLTQC	VHVKYIPLHS	TVELIMFDDH	IFHLHGYSFY	VTDVRQMDTK
AfMCORP	VLNVNNATFT	YPSSPLLSQC	VHVRYIPLGA	TVEIILLDDL	VYHLHGYTFY	VVGARKFGRS
DpMCORP	IYRINNLTFS	YPASPLLTQC	VHLEAVDLGT	SVEIMIVEEH	ILHFHGNHFY	IVGSRQFERP

	....|....|	....|....|	....|....|	....|....|	....|....|	....|....|
	425	435	445	455	465	475
AgMCO1	TTIEDIRRMD	EAPIKDTVTI	PDGGYTIIRF	IANNPGYWLF	H-CHIEFHAE	IGMSLVLKVG
AgMCO2A	SNVKKINLKH	APPLKDTIAV	PNNGYVVLRF	RADNPGFWLF	H-CHFLFHIV	IGMNLILQVG
AgMCO3	PITLEIAQNI	APPRKDTVSI	PSRGYARVRF	RADNPGFWLM	H-CHYEWHTA	VGMALVLQVG
AgMCO4	FGTQSEKVDF	VPPYKDTVSV	PSRGYTRIRF	RADNPGFWLV	H-CHFEWHLG	IGMSFVLQVG
AgMCO5	ILT--DQIAY	LPPYKDTQSI	PNRGYVRIRF	RADNPGFWLV	H-CHFEWHLA	DGMGLVLQIG
AaAAEL007802	ITAEEVKRLD	EAPIKDTVTI	PDGGYTIIRF	IANNPGYWLF	H-CHIEFHAE	IGMSLVLKVG
AaAAY29698	SNVKKINLKH	APPLKDTIAV	PNNGYVVLRF	RADNPGFWLF	H-CHFLFHIV	IGMNLILQVG
AaAAEL001667	PITMERAQKI	APPKKDTVSV	PSKGYTRVRF	VADNPGFWLM	H-CHYEWHTA	VGMVLVLQVG
AaAAEL001632	IKHSSEKLRW	IPPYKDTVSI	PSRGYTKIRF	RADNPGFWLV	H-CHFEWHLG	IGMSFILQVG
AaAAEL001640	AIN--SRLKY	LPPFKDTISI	PNEGFVKTRF	RASNPGFWFV	H-CHFEWHLG	TGMGLVLQVG
AaAAEL001672	AKT--LRLKY	LPPYKDTISI	PNEGYVKVRF	RANNAGFWLV	H-CHFEWHLG	TGMGLVLQVG
AmLOC724890	VTVDKVKALD	KAPLKDTVTV	PDGGYTVVRF	HANNPGYWLF	H-CHIEFHAE	VGMSLIFKVG
AmLOC410365	KNVKKINLKH	APPAKDTIAV	PNNGYVIFRF	RADNPGYWLF	H-CHFLFHIL	IGMNLILHVG
AmLOC552811	RNISRQDINE	VPPGKDTAKI	PMGGYVIVRF	KADNPGWWLL	H-CHFSWHHI	TGMELVILVG
DmMCO1	VTVEMIKQLD	QPPVKDTVTI	PDGGYTIIRF	EASNPGYWLF	H-CHIEFHAE	IGMALVFKVG
DmLac2	SSVKKINLKH	APPTKDTIAV	PNNGYVVLRF	RADNPGFWLF	H-CHFLFHIV	IGMNLILQVG
DmMCO3	NDLRNIQELD	RAVAKDTVQI	PGQGYIIVRF	ISNNPGFWLY	H-CHVEAHAV	QGMVAVLKIG
DmCG32557	-KIGQIEQID	KAPLKDSVQV	PAFGYTILRF	YSNSPGYWMF	H-CHISPHSE	NGMAAVVRVG
BmMCO1	TTIEEVKAFD	EAPIKDTVTV	PDGGYTVIRF	KADNPGYWLF	H-CHIEFHVE	VGMALVFKVG
BmLac2	QNVKKINLKH	APPAKDTIAV	PNNGYVILRF	RATNPGFWLL	H-CHFLFHIV	IGMSLVLQVG
MsLac1	TTIEEIKAFD	EAPIKDTVTV	PDGGYTVIRF	KADNPGYWLF	H-CHIEFHVE	VGMALVFKVG
MsLac2	QNVKKINLKH	APPAKDTIAV	PNSGYVILRF	RATNPGFWLL	H-CHFLFHIV	IGMSLVLQVG
TcLac1	VNVSEILKMD	QAPLKDTVTV	PDGGFTIIRF	KATNPGYWLF	H-CHIEFHVE	VGMALVFKIG
TcLac2A	QNVKKINLKH	APPAKDTIAV	PNNGYVVLRL	RANNPGFWLF	H-CHFLFHIV	IGMNLVLQVG
ApLOC100165676	TTVEEIEQMD	RAPLKDTVTV	PDGGFTILRF	LADNPGYWLF	H-CHIEFHVE	VGMATVFKIG
ApLOC100164049	KNVKKINLKH	APPLKDTIAV	PNNGYVVFRF	RADNPGYWLF	H-CHFLFHIV	IGMNLVLHVG
PhPHUM024710	TSEDLVRRLD	KPPIKDTVTT	PDGGYSILRF	HAINPGYWMF	H-CHIDFHVE	MGMVLLFKVG
PhPHUM554290	RNIKKINLKH	APPAKDTIAV	PNNGYVVLRF	RADNPGYWLF	H-CHFLFHIV	IGMNLVVHVG
AgMCORP	PGTALGALGG	QPLQRDTIVV	RRGSTVAVRF	VANLAGLWLL	RDIGSP-GWS	RGLDVVLSVG
TcMCORP	PSTDEIKLLD	QPAIKDTIRI	PKNSVVALRF	LADNPGFWML	R-DEGSRGWT	RGLDIVLQVG
AaMCORP	ENWQTQLLN-	-PVLLDTVRI	ESNSMVVLRF	VASNAGLWML	RDLDAEHEWS	RGLDVLLNVR
CqMCORP	EGWQHQQLDR	LPVRRDTVRI	GRRSLVVLRF	VASHAGLWLL	RDLGAEHGWS	RGLDVLLVVH
BmMCORP	LSKETVIKMN	EPVLKDTIVI	PKFGVVALRF	KADNPGYWMM	R-DERSTHWT	RGLDFILKVG
MsMCORP	LAKEDVIKMN	EPVIKDTIVI	PKFGAVSLRF	KADNPGYWMM	R-DERSTHWT	RGLDFILKVG
CfMCORP	VSLQEVKKLD	KPPIKDTVIV	PKFGVVALRF	KADNPGYWML	R-DEHAADWT	RGLDVVLQVG
MrMCORP	VSLQELKSLD	DTVAKDTVVV	PKFGAVAIRF	KANNPGYWML	R-DEHSPYWT	RGLDVILQVG
PhMCORP	VATKEQIKQF	DPVVKDTLAL	SSGYLYVIRF	KADNPGYWLF	D-EENSSHFS	KGLSLVFKVG
BtMCORP	VSLHELKSLD	DTVAKDTVVV	PKFGAVALRF	KADNPGYWML	R-DEHAAEWT	RGLDVILQVG
BiMCORP	VSLQELKSLD	DTVAKDTVVV	PKFGAVALRF	KADNPGYWML	R-DEHAAEWT	RGLDVILQVG
ApMCORP	KSADHAKKLD	SSVLKNTVVV	PAAGVSVVRF	IADNPGYWLF	R-SEKTSEWS	SGLSLIFRVS
HsMCORP	MSLQNIRNLD	KAPAKDTIVV	PKFGAVALRF	KADNPGYWML	R-DEHAADWT	RGLDVILQVG
DpMCORP*	LEKETVMKMN	QPVRKDTIVI	PKFGVVAALRF	KADNPGYWMM	R-DERSAHWT	RGLDFVLKVG
AfMCORP	VSLQELKSMD	DTVAKDTVVV	PKFGAVALRF	KANNPGYWML	R-DEHAAEWT	RGLDVILQVG
DpMCORP	MSRKEIRELD	QPVLKDTIRI	PRFGVVILRF	LAKNPGIWML	R-DENSHGWS	KGLDLAIEVG

	....|....| ..	
	485	
AgMCO1	EMLPAPANFP	TC
AgMCO2A	DLPPVPPNFP	TC
AgMCO3	EMVKAPADFP	KC
AgMCO4	EMKQAPKDFP	RC
AgMCO5	EMLKPPANFP	RC
AaAAEL007802	EMVAAPHNFP	TC
AaAAY29698	DLPPVPPNFP	TC
AaAAEL001667	SFVKPPAGFP	TC
AaAAEL001632	QMIKTPPGFP	TC
AaAAEL001640	QMLKAPPGFP	RC
AaAAEL001672	QMLKAPPDFP	RC
AmLOC724890	DMLPVPRNFP	LC
AmLOC410365	DLPPIPPNFP	RC
AmLOC552811	DLPPIPKNFP	KC
DmMCO1	QMVPVPENFP	TC
DmLac2	DLPPVPPGFP	TC
DmMCO3	QMKNIPARVR	C-
DmCG32557	EMKMCPVSN-	C-
BmMCO1	DMPPVPREFP	KC
BmLac2	DLPPVPPNFP	TC
MsLac1	DMAPLPRDFP	TC
MsLac2	DLPPVPPGFP	TC
TcLac1	EMPPVPKDFP	QC
TcLac2A	DLPPVPPNFP	TC
ApLOC100165676	EMPPPPPGFP	KC
ApLOC100164049	DLPPVPENFP	RC
PhPHUM024710	MFKLPPDNFP	KC
PhPHUM554290	DLPPVPPNFP	RC
AgMCORP	PQPDIPRNFP	AC
TcMCORP	DMVSTPTDFP	TC
AaMCORP	TEINFPDDFP	KC
CqMCORP	DEFDIPGDFP	TC
BmMCORP	DLVQAPPDFP	KC
MsMCORP	DFVQAPADFP	KC
CfMCORP	DMIPPPQDFP	KC
MrMCORP	DMASAPQDFP	KC
PhMCORP	DFPNVPDNFP	KC
BtMCORP	DMVPAPEDFP	KC
BiMCORP	DMVPAPEDFP	KC
ApMCORP	SFPQVPEDFP	KC
HsMCORP	DMVAAPQDFP	KC
DpMCORP*	DFVKAPADFP	KC
AfMCORP	DMVPAPEDFP	KC
DpMCORP	QMVSTPSNFP	TC

Figure S1. Alignment of Insect MCORPs and MCOs for Phylogenetic Analysis. The amino-terminal and carboxyl-terminal ends of sequences, which are highly variable, were left out of the alignment. Sequences beginning with the cysteine rich region (11) were aligned by ClustalW in MEGA5 and adjusted by eye. Gaps were omitted in the alignment for phylogenetic analysis. Abbreviations used are: Ag, *Anopheles gambiae*; Aa, *Aedes aegypti*; Cq, *Culex quinquefasciatus*; Tc, *Tribolium castaneum*; Dp, *Dendroctonus ponderosae*; Ms, *Manduca sexta*; Bm, *Bombyx mori*; Dp*, *Danaus plexippus*; Cf, *Camponotus floridanus*; Hs, *Harpegnathos saltator*; Bt, *Bombus terrestris*; Bi, *Bombus impatiens*; Mr, *Megachile rotundata*; Af, *Apis florae*; Ap, *Acyrthosiphon pisum*; Ph, *Pediculus humanus corporis*; Dm, *Drosophila melanogaster*; Am, *Apis mellifera*.
