# Supplementary material for: A Multicopper Oxidase-Related Protein Is Essential for Insect Viability, Longevity and Ovary Development
Source: PLoS One. 2014 Oct 20;9(10):e111344. doi: 10.1371/journal.pone.0111344 (PMC4203857; doi:10.1371/journal.pone.0111344)
Supplement: Table S1 — Primers used in this work. (DOCX) [file pone.0111344.s003.docx]

**Table S1. Primers used in this work**

| Primer name | Primer sequence (5’ – 3’) | Primer usage |
| --- | --- | --- |
| TcMCORP-F | GGATCCCCAGAGGGCACATTC | Cloning of TcMCORP to generate antiserum |
| TcMCORP-R | GTCGACTTACATCAGAAAGAAATC |  |
| RACE 5’ P | AGGTCCTGCGGGCACAGCATCGTTT | 5’ RACE of AgMCORP |
| RACE 5’ nested P | AACTCCTCCGACCGATGGGGAAACG |  |
| RACE 3’ P | GGACGGCTTGCGGTGAAACGATGCT | 3’ RACE of AgMCORP |
| RACE 3’ nested P | CAAAGGATCTGCAGCGCGGGTACGA |  |
| fAgMCORP-F | GCGTATGCGAGTGCTGCTA | Cloning of full length AgMCORP |
| fAgMCORP-R | CAACTACTACCACCGCCACC |  |
| AgMCORP-F | GAATTCGTTTTGATACACTTT | Cloning of AgMCORP to generate antiserum |
| AgMCORP-R | GTCGACTTAAATTAAAAAGTACCT |  |
| tAgMCORPS2-F | AGATCTGCCGTTTCGTGCGAC | Cloning of truncated AgMCORP into pMT/Bip/V5-His A vector |
| tAgMCORPS2-R | GAATTCTTAAATTAAAAAGTACCT |  |
| fAgMCORPS2-F | ACTAGTATGAAAACACTGCAG | Cloning of full length AgMCORP into pMT/V5-His A vector |
| fAgMCORPS2-R | GAATTCTTAAATTAAAAAGTA |  |
| TcMCORPSf9-F | GTCGACATGAAGTTATGCATA | Cloning of Bip-TcMCORP to make recombinant protein |
| TcMCORPSf9-R | GAATTCTTACATCAGAAAGAAATC |  |
| TcRTPCR-F | GCTCCAAAGACCGTGTA | RT-PCR of TcMCORP in developmental stages  / Test the efficiency of RNAi |
| TcRTPCR-R | TCTTGGCTCGCATCTTG |  |
| TcRPS6-F | AGATATATGGAAGCATCATGAAGC | RT-PCR of TcRPS6 |
| TcRPS6-R | CGTCGTCTTCTTTGCTCAAATTG |  |
| qTcMCORP-F | CCAATGTCCAATACTTAGC | RT-PCR of TcMCORP in tissues |
| qTcMCORP-R | GCCTGATAGAGATGTGTT |  |
| qRPS3-F | GCAGAGTCTCTTAGATTCA | RT-PCR of TcRPS3 |
| qRPS3-R | ATTCCATGATGTACCTCAA |  |
| AgRTPCR-F | CCTTCAGTCAGTCGCATCCT | RT-PCR of AgMCORP |
| AgRTPCR-R | TCTCGTGGGTCGGTTCTCC |  |
| AgRPS7-F | CGCTATGGTGTTCGGTTCC | RT-PCR of AgRPS7 |
| AgRPS7-R | TGCTGCAAACTTGGGCTAT |  |
| dsRNATc-F | TAATACGACTCACTATAGGGAG  GGTTTGTTTGGTGCTCT | Synthesis of TcMCORP dsRNA |
| dsRNATc-R | TAATACGACTCACTATAGGGAG  ACTTTGCCTATTTGAGA |  |
| dsRNAVer-F | TAATACGACTCACTATAGGG  GTCTTGGTGGA | Synthesis of TcVer dsRNA |
| dsRNAVer-R | TAATACGACTCACTATAGGG  CCGCCATTTC |  |
| dsRNAAg-F | TAATACGACTCACTATAGGGAG  ATTCCAGGCGGACAGGG | Synthesis of AgMCORP dsRNA |
| dsRNAAg-R | TAATACGACTCACTATAGGGAG  TGCGGTGTAAAGCGAAT |  |
| dsRNAGFP-F | TAATACGACTCACTATAGGGCGATGC | Synthesis of GFP dsRNA |
| dsRNAGFP-R | TAATACGACTCACTATAGGGCGGACT |  |
| qAgMCORP-F | CATTACACTGCACTGGAC | qPCR of AgMCORP |
| qAgMCORP-R | TGGAATTTGTACTGGAACC |  |
| qRPS7-F | GTGGTCGGCAAGCGTATCC | qPCR of AgRPS7 |
| qRPS7-R | GGTGGTCTGGTTCTTATCC |  |
