# Supplementary material for: A Multicopper Oxidase-Related Protein Is Essential for Insect Viability, Longevity and Ovary Development
Source: PLoS One. 2014 Oct 20;9(10):e111344. doi: 10.1371/journal.pone.0111344 (PMC4203857; doi:10.1371/journal.pone.0111344)
Supplement: Table S2 — NCBI accession numbers of sequences used for phylogenetic analysis. (DOCX) [file pone.0111344.s004.docx]

**Table S2. NCBI accession numbers of sequences used for phylogenetic analysis**

| Species | Phylogenetic group | NCBI accession number |
| --- | --- | --- |
| *Anopheles gambiae* | MCO1 | AAN17505 |
| *A. gambiae* | MCO2A | AAX49501 |
| *A. gambiae* | MCO3 | ABQ95972 |
| *A. gambiae* | MCO4 | ABY84643 |
| *A. gambiae* | MCO5 | ABY84644 |
| *A. gambiae* | MCORP | KJ500312 |
| *Aedes aegypti* | AAEL007802 | XP_001652917 |
| *A. aegypti* | AAY29698 | AAY29698 |
| *A. aegypti* | AAEL001667 | XP_001653727 |
| *A. aegypti* | AAEL001632 | XP_001653728 |
| *A. aegypti* | AAEL001640 | XP_001653729 |
| *A. aegypti* | AAEL001672 | XP_001653730 |
| *A. aegypti* | MCORP | XP_001652083 |
| *Culex quinquefasciatus* | MCORP | XP_001862832 |
| *Drosophila melanogaster* | MCO1 | NP_609287 |
| *D. melanogaster* | Lac2 | NP_724412 |
| *D. melanogaster* | MCO3 | NP_651441 |
| *D. melanogaster* | CG32557 | NP_573249 |
| *Manduca sexta* | Lac1 | AAN17506 |
| *M. sexta* | Lac2 | AAN17507 |
| *M. sexta* | MCORP | Msex2.04321* |
| *Bombyx mori* | MCO1 | DAA06286 |
| *B. mori* | Lac2 | NP_001103395 |
| *B. mori* | MCORP | XP_004930412 |
| *Danaus plexippus* | MCORP | EHJ73418 |
| *Camponotus floridanus* | MCORP | EFN64969 |
| *Harpegnathos saltator* | MCORP | EFN82577 |
| *Apis mellifera* | LOC724890 | XP_001120790 |
| *A. mellifera* | LOC410365 | XP_006562317 |
| *A. mellifera* | LOC552811 | XP_625189 |
| *Apis florea* | MCORP | XP_003695203 |
| *Bombus terrestris* | MCORP | XP_003399857 |
| *Bombus impatiens* | MCORP | XP_003486821 |
| *Megachile rotundata* | MCORP | XP_003707167 |
| *Tribolium castaneum* | Lac1 | NP_001034514 |
| *T. castaneum* | Lac2A | NP_001034487 |
| *T. castaneum* | MCORP | KJ500311 |
| *Dendroctonus ponderosae* | MCORP | ENN80477 |
| *Pediculus humanus corporis* | PHUM024710 | XP_002422943 |
| *P. h. humanus* | PHUM554290 | XP_002431869 |
| *P. h. humanus* | MCORP | XP_002423995 |
| *Acyrthosiphon pisum* | LOC100165676 | XP_001948070 |
| *A. pisum* | LOC100164049 | XP_001950788 |
| *A. pisum* | MCORP | XP_001946224 |

*Accession number is from Manduca Base (http://agripestbase.org/manduca/)
